# An evidence-informed psychosocial intervention program for caregiver burden among family caregivers of children with autism spectrum disorder: a multi-stage development and Delphi study

**DOI:** 10.1371/journal.pone.0354823

**Published:** 2026-08-12

**Authors:** Xinyu Tian, Wenna Qian, Jiashuo Zhang, Jialin Li, Biyun Chen, Xiaohan Li, Xiuchuan Yan

**Affiliations:** 1 School of Nursing, Tianjin Medical College, Tianjin, China; 2 School of Nursing, China Medical University, Shenyang, China; 3 The Second Affiliated Hospital of Dalian Medical University, Dalian, China; 4 Department of Pediatrics, Shenzhen Hospital, Beijing University of Chinese Medicine, Shenzhen, China; 5 Haihe Hospital, Tianjin University, Tianjin, China; UTM Skudai: Universiti Teknologi Malaysia, MALAYSIA

## Abstract

**Background:**

Family caregivers of children with autism spectrum disorder (ASD) often experience substantial and multidimensional caregiver burden, which may adversely affect their physical and psychological well-being, family functioning, and the child’s rehabilitation. Although caregiver-focused psychosocial interventions have shown promise, structured and theory-informed programs specifically designed to address caregiver burden in this population remain limited.

**Methods:**

A multi-stage intervention development design was used, guided by an integrated framework of the ABC-X family stress model and stress and coping theory. First, best-available evidence was synthesized to identify relevant intervention strategies. Second, findings from prior quantitative and qualitative phases of a mixed-methods research program were integrated to define intervention targets and draft content. Third, the draft program was refined through a two-round asynchronous modified Delphi consultation to improve content relevance, clarity, and implementation planning.

**Results:**

The finalized program comprised three core modules (stressors, cognitive appraisal, and coping), seven themes, and 22 components. It incorporated psychoeducation, mindfulness-based approaches, gratitude-based expressive writing, acceptance and commitment-informed strategies, self-compassion training, and problem-solving skills. Expert response and authority were high; however, Kendall’s W values were modest across the two rounds (0.23 and 0.25). These findings suggest that the Delphi process contributed to systematic refinement and expert appraisal of the program and reflected experts’ perceptions of the relevance, clarity, and appropriateness of its content; practical feasibility remains to be tested directly with caregivers.

**Conclusions:**

This study developed a theory-driven and evidence-informed psychosocial intervention framework for caregiver burden among family caregivers of children with autism spectrum disorder. The program provides a structured basis for ongoing caregiver-informed feasibility testing and subsequent effectiveness evaluation; its acceptability, delivery fidelity, and later effectiveness require further empirical validation.

## Introduction

Autism spectrum disorder (ASD) is widely recognized as a major public health concern because of its long-term impact on affected individuals, families, and health and social care systems [1,2]. The World Health Organization estimated that approximately 1 in 127 people globally had autism in 2021, while emphasizing substantial variation across studies and settings [3]. Epidemiological data from multiple regions also indicate marked increases in identified prevalence over recent decades, resulting in a growing population of children who require sustained care and support across developmental stages [4–6]. In China, limited diagnostic resources, regional disparities in service accessibility, and under-identification may further contribute to an underestimation of the true prevalence of ASD, while shifting a substantial and often unrecognized caregiving burden onto families [6].

ASD is a lifelong neurodevelopmental condition characterized by persistent difficulties in social communication and restricted, repetitive patterns of behavior [7,8]. Because children with ASD often require long-term and coordinated support, family caregivers frequently assume primary responsibility for managing daily care, coordinating therapeutic services, and making ongoing care-related decisions [9]. These responsibilities place family caregivers at the center of care delivery and expose them to sustained psychosocial strain.

Family caregivers of children with ASD consistently report higher levels of caregiver burden than caregivers of children with other chronic conditions or developmental disabilities [10–12]. In this population, caregiver burden is commonly understood as a multidimensional construct involving physical exhaustion, emotional distress, social restriction, time dependency, and limitations in personal development [9]. Elevated caregiver burden has been associated with anxiety, depressive symptoms, and poorer quality of life, which may in turn undermine caregivers’ capacity to sustain long-term caregiving and to support children’s rehabilitation and developmental needs [13–15].

Despite increasing recognition of caregiver burden as a critical issue in ASD care, current interventions remain limited in their capacity to address caregivers’ complex and interrelated needs in a coherent manner. Recent reviews have shown that interventions for families of children with ASD are often fragmented and tend to focus on isolated psychological outcomes or short-term symptom relief, rather than addressing the broader psychosocial determinants of caregiver burden through a structured program [16,17]. This fragmentation may limit their integration into routine services and reduce their sustainability and scalability.

Existing interventions for families of children with ASD are commonly categorized as caregiver-mediated or caregiver-focused. Caregiver-mediated interventions primarily seek to improve child outcomes by training caregivers to deliver therapeutic strategies [16]. Although these approaches may benefit child functioning, they may also intensify caregiving demands and increase caregiver stress and burden [14,17]. In contrast, caregiver-focused interventions aim to support caregivers’ own psychological well-being, coping capacity, and adjustment, and appear more directly relevant to reducing caregiver burden [16,17]. However, substantial heterogeneity remains in intervention content, delivery formats, and outcome measures, highlighting the need for a more integrated and theory-informed program.

**Fig 1:**
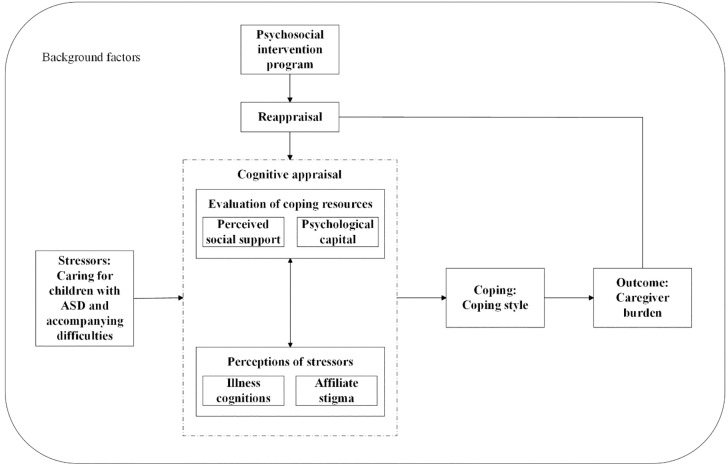
Theoretical framework underpinning the development of the psychosocial intervention program for caregiver burden among family caregivers of children with ASD.

To guide intervention development, the present study integrated the ABC-X family stress model and Lazarus and Folkman’s stress and coping theory [18,19]. Within this framework, caregiver burden can be understood as the product of interactions among family caregiving stressors, caregivers’ appraisal of those stressors and available resources, and their coping responses. This scope differs from the job demand-control-support model, which was developed to explain strain arising from paid work demands, decision latitude, and workplace support [20]. Although occupational models are valuable for employed populations, the integrated family-stress and coping framework was selected because it more directly represents unpaid family caregiving, illness-related meaning-making, family resources, stigma, and coping processes. It therefore provides a closer conceptual fit for a caregiver-focused intervention targeting multiple interrelated domains rather than isolated symptoms (Fig 1).

Accordingly, this study aimed to develop and refine an evidence-informed psychosocial intervention program for family caregivers of children with ASD. Using a multi-stage intervention development approach, the study integrated findings from a best evidence synthesis, prior quantitative and qualitative phases of a mixed-methods research program, and Delphi expert consultation to construct a theoretically grounded program for subsequent feasibility and effectiveness testing.

## Methods

### Study design

This study employed a multi-stage, evidence-informed design to develop a psychosocial intervention program targeting caregiver burden among family caregivers of children with ASD. The development process comprised three sequential phases: (1) best evidence synthesis; (2) program conceptualization through integration of theoretical frameworks and empirical findings from prior quantitative and qualitative phases of a mixed-methods research program; and (3) a two-round asynchronous modified Delphi consultation to refine the program content and implementation plan. Reporting of the consensus component was guided by the ACCORD (ACcurate COnsensus Reporting Document) guideline [21].

### Phase 1. Best evidence synthesis

#### Formulation of the research question.

A best evidence synthesis was conducted to inform the development of the intervention program. The review question was formulated using the PIPOST framework, which specified the following elements: population (family caregivers of children with ASD); intervention (strategies targeting caregiver burden and its related intervention targets); professionals (clinical healthcare providers); outcomes (alleviation of caregiver burden); settings (hospital or home); and types of evidence (clinical practice guidelines, evidence summaries, systematic reviews, and expert consensus documents). The best evidence synthesis was registered with the Shanghai Evidence-Based Nursing Center (Registration No. ES20245306).

#### Literature search.

Guided by the 6S evidence hierarchy, literature was searched from the top down across evidence-based resources, guideline databases, evidence summary databases, systematic review databases, and comprehensive bibliographic databases. The search period covered database inception to September 15, 2024. Search terms were developed in both English and Chinese. Boolean operators were used, and both subject headings and free-text terms were applied. Detailed search strategies are presented in S1 File.

#### Study selection.

Eligibility criteria were developed according to the PIPOST framework. The inclusion criteria were as follows: (1) family caregivers of children with ASD aged ≥18 years; (2) interventions targeting caregiver burden or related intervention targets; (3) outcomes including at least caregiver burden or parenting/parental stress; (4) hospital or home settings; (5) evidence types including clinical practice guidelines, evidence summaries, systematic reviews, and expert consensus documents; and (6) publications in English or Chinese. The exclusion criteria were as follows: (1) full text unavailable; (2) interventions or outcomes not extractable; and (3) duplicate publications. Retrieved records were imported into EndNote for deduplication using both software-assisted and manual methods. Two reviewers independently screened titles and abstracts in two consecutive rounds, followed by full-text screening of retained records. Disagreements were resolved through discussion within the research team.

#### Quality appraisal.

Two reviewers with relevant clinical experience and formal training in evidence-based nursing independently appraised the methodological quality of the included literature. Disagreements were resolved through discussion within the research team. Appraisal tools were selected according to the type of evidence. For evidence summaries, methodological quality was assessed by tracing the original references. The Appraisal of Guidelines for Research and Evaluation II (AGREE II) instrument was used for clinical practice guidelines. The Joanna Briggs Institute (JBI) critical appraisal tools (2016 version) were used for systematic reviews and expert consensus documents.

#### Evidence extraction, synthesis, and grading.

Two reviewers independently extracted, synthesized, and graded the evidence. Disagreements were resolved through discussion within the research team. For evidence with consistent content, concise evidence statements were extracted. For complementary evidence, findings were integrated according to logical consistency. For conflicting evidence, priority was given to evidence-based sources, higher-quality evidence, and more recent evidence. According to the research design types of the original studies underpinning the included evidence, evidence was graded from Level 1 to Level 5 using the JBI 2014 pre-grading system, with Level 1 representing the highest level and Level 5 the lowest.

### Phase 2. Program conceptualization and draft formulation

The preliminary intervention program was formulated through an iterative process integrating the theoretical framework, empirical inputs from the prior quantitative and qualitative phases, and recommendations from the best evidence synthesis. No primary data from the earlier phases were re-analysed in the present study. Their relevance was assessed according to population and setting correspondence, temporal proximity, conceptual alignment with the intervention framework, and convergence with the evidence synthesis. Methodological confidence was judged from the design-specific safeguards used in each phase, including standardized multicentre procedures and validated measures in the quantitative study and maximum-variation sampling, reflexivity, team-based analysis, participant feedback, and an audit trail in the qualitative study. Because these phases were components of the same research program rather than external evidence studies selected for the review, they were not assigned separate external quality-appraisal scores.

#### Theoretical foundation of the intervention program.

A theoretical framework for understanding caregiver burden among family caregivers of children with ASD was established by integrating the ABC-X family stress model with stress and coping theory. This framework informed three core modules: stressors, cognitive appraisal, and coping. Cognitive appraisal was operationalized as two related evaluative processes: appraisal of stressors and appraisal of available coping resources. Appraisal of stressors included illness cognition and affiliate stigma, whereas appraisal of available coping resources included psychological capital and perceived social support. Both processes were placed within the Cognitive Appraisal module because they represent evaluative judgments that precede and shape coping responses. Coping referred to the behavioral and cognitive strategies adopted in response to caregiving demands. Accordingly, psychological capital, perceived social support, illness cognition, affiliate stigma, and coping strategies were identified as key intervention targets (Fig 1).

#### Contribution of quantitative findings to intervention development.

The prior quantitative phase was a multicentre cross-sectional survey conducted from January to April 2024 in four rehabilitation centres in China. Using convenience sampling, 320 family caregivers completed the survey and 307 valid responses were analysed. Validated measures, standardized site procedures, confirmatory factor analysis, multiple regression, structural equation modelling, and bias-corrected bootstrap analyses were used to examine caregiver burden and its psychosocial pathways. This phase is available as a preprint and has not been treated as peer-reviewed evidence in the present study [22]. Psychological capital, perceived social support, illness cognition, affiliate stigma, and coping tendency showed direct and/or indirect associations with caregiver burden and were therefore selected as intervention targets. Caregivers of an only child with ASD, female caregivers, unemployed caregivers, and caregivers of children with greater ASD severity were identified as priority populations for later intervention targeting and recruitment.

#### Contribution of qualitative findings to intervention development.

The prior qualitative phase used descriptive phenomenology and purposive maximum-variation sampling. Sixteen family caregivers from the same four rehabilitation centres participated in face-to-face semi-structured interviews between May and August 2024. Data were analysed using Colaizzi’s method, with reflexive journaling, team-based analysis, participant feedback, and an audit trail used to support trustworthiness; reporting followed COREQ. Caregivers described the causes and influencing factors of burden, different appraisal and coping patterns, expectations regarding illness prognosis, and preferences concerning intervention content, format, timing, and personnel. These findings informed the draft program and represented indirect caregiver contribution to its development. However, caregivers did not directly review, co-design, or validate the finalized program after Delphi refinement. The integrated program outline based on the theoretical framework and findings from the quantitative and qualitative phases is presented in Table 1.

**Table 1:** Integrated program outline based on the theoretical framework and findings from the quantitative and qualitative phases.

Intervention module	Intervention theme	Source of theme
Stressors	Stressors experienced by family caregivers	Qualitative findings
Cognitive appraisal	Expectations regarding illness prognosis	Qualitative findings
Cognitive appraisal	Psychological capital	Theoretical framework; quantitative findings
Cognitive appraisal	Perceived social support	Theoretical framework; quantitative findings
Cognitive appraisal	Illness cognition	Theoretical framework; quantitative findings
Cognitive appraisal	Affiliate stigma	Theoretical framework; quantitative findings
Coping	Coping strategies	Theoretical framework; quantitative findings

#### Evidence-informed intervention strategies.

The best evidence synthesis provided specific intervention strategies and content to support program development. The evidence recommended the use of two or more core intervention components to address caregiver burden [23,24]. Based on the synthesized evidence, mindfulness-based interventions were aligned with psychological capital [23,25]; gratitude-based interventions combined with expressive writing were aligned with perceived social support [24]; acceptance and commitment therapy was aligned with illness cognition [16,26]; self-compassion practices were aligned with affiliate stigma [27]; and problem-solving skills training was aligned with coping strategies [16]. These themes were therefore matched with corresponding intervention strategies in the preliminary program outline, and the detailed intervention content is presented in Table 2. In addition, evidence supporting the provision of accurate and reliable information about ASD led to the inclusion of “basic knowledge of ASD” as an additional intervention theme [25]. The evidence synthesis also informed decisions regarding intervention personnel, delivery format, and intervention setting [16,28].

**Table 2:** Preliminary outline of the psychosocial intervention program for caregiver burden among family caregivers of children with ASD.

Intervention module	Intervention theme	Intervention content
1. Stressors	1.1 Basic knowledge of ASD	1.1.1 Knowledge of ASD etiology
		1.1.2 Clinical manifestations
		1.1.3 Diagnosis and treatment
	1.2 Stressors experienced by family caregivers	1.2.1 Caregiving tasks and associated caregiving difficulties
2. Cognitive appraisal	2.1 Expectations regarding illness prognosis	2.1.1 Introduction to knowledge of illness prognosis
		2.1.2 Sharing expectations regarding illness prognosis
	2.2 Psychological capital	2.2.1 Overview of mindfulness
		2.2.2 Mindful breathing
		2.2.3 Body scan
		2.2.4 Mindful eating
		2.2.5 Mindful stretching
		2.2.6 Mindful sitting
		2.2.7 Emotional awareness
		2.2.8 Mindful walking
	2.3 Perceived social support	2.3.1 Gratitude-based intervention
		2.3.2 Expressive writing
	2.4 Illness cognition	2.4.1 Overview of acceptance and commitment therapy (ACT)
		2.4.2 Acceptance and cognitive defusion training
		2.4.3 Values clarification
		2.4.4 Values-based committed action
		2.4.5 Advanced acceptance and cognitive defusion training
		2.4.6 Self-observation
	2.5 Affiliate stigma	2.5.1 Overview of self-compassion
		2.5.2 Self-compassion meditation
		2.5.3 Self-compassion dialogue
3. Coping	3.1 Coping strategies	3.1.1 Problem-solving skills

#### Integration and structuring of the intervention program.

Integration of the theoretical framework, quantitative and qualitative findings, and evidence-informed intervention strategies resulted in a preliminary program outline comprising three core modules, eight intervention themes, and 26 specific components. Based on this outline, a preliminary draft of the intervention program was developed and subsequently submitted for refinement through Delphi expert consultation.

### Phase 3. Delphi expert consultation

#### Expert selection.

A purposive sampling strategy was employed to recruit experts from relevant disciplines, including autism treatment, psychological therapy, psychiatric nursing, clinical nursing, and nursing education. The inclusion criteria were as follows: (1) a bachelor’s degree or above; (2) an associate senior professional title or above, or an equivalent senior academic or clinical rank; (3) at least 10 years of professional or research experience in the relevant field; and (4) willingness to participate in the study. A total of 16 experts were included in the Delphi consultation. This panel size was consistent with methodological recommendations that Delphi studies generally include 15–30 experts [29].

#### Delphi procedure.

After obtaining the experts’ agreement to participate, a two-round asynchronous modified Delphi consultation was conducted. Experts were recruited between 01/01/2025 and 28/02/2025. Structured questionnaires were distributed electronically by email or WeChat. The first round was administered between 01/01/2025 and 31/01/2025, and the second between 15/02/2025 and 28/02/2025. After round 1, quantitative ratings and anonymized written comments were summarized. The research team revised the program through documented discussion and used the revised program and questionnaire in round 2. The process ended after two rounds because no further structural changes were indicated, all retained items met the predefined item-level criteria, and an additional round was not expected to resolve the remaining multidisciplinary variation [30,31].

#### Data analysis.

Quantitative data were analyzed using SPSS version 26.0 (IBM Corp., Armonk, NY, USA). Delphi indicators included expert response rate, expert authority coefficient (Cr), mean item importance score, coefficient of variation (CV), and Kendall’s coefficient of concordance (W). The authority coefficient was calculated as the mean of the judgment basis coefficient (Ca) and familiarity coefficient (Cs). Items were retained, modified, merged, or deleted using predefined thresholds for importance and variation together with written comments. Divergent recommendations were reconciled by the research team through comparison with the theoretical framework, prior empirical findings, best evidence synthesis, clinical feasibility, and internal coherence. Decisions and reasons were documented between rounds; no item was retained or removed solely on the basis of Kendall’s W. Detailed thresholds and procedures are provided in S2 File.

#### Ethical considerations.

This study was approved by the Ethics Committee of Shenzhen Hospital, Beijing University of Chinese Medicine (Approval No. KY-2024–368). Before participation, all experts were fully informed about the study aims and procedures, and written informed consent was obtained from all experts prior to participation. All expert information was kept confidential and was not disclosed publicly without authorization.

#### Use of artificial intelligence tools.

During manuscript revision, OpenAI ChatGPT was used to support English-language editing, consistency checking, and revision-related wording refinement. No AI tool was used to generate or analyse primary data, conduct statistical analyses, create figures or tables, select references, or make scientific interpretations. All AI-assisted text was critically reviewed and revised by the authors, who take full responsibility for the final content of the manuscript.

## Results

### Phase 1. Best evidence synthesis

#### Evidence identification and selection.

**Fig 2:**
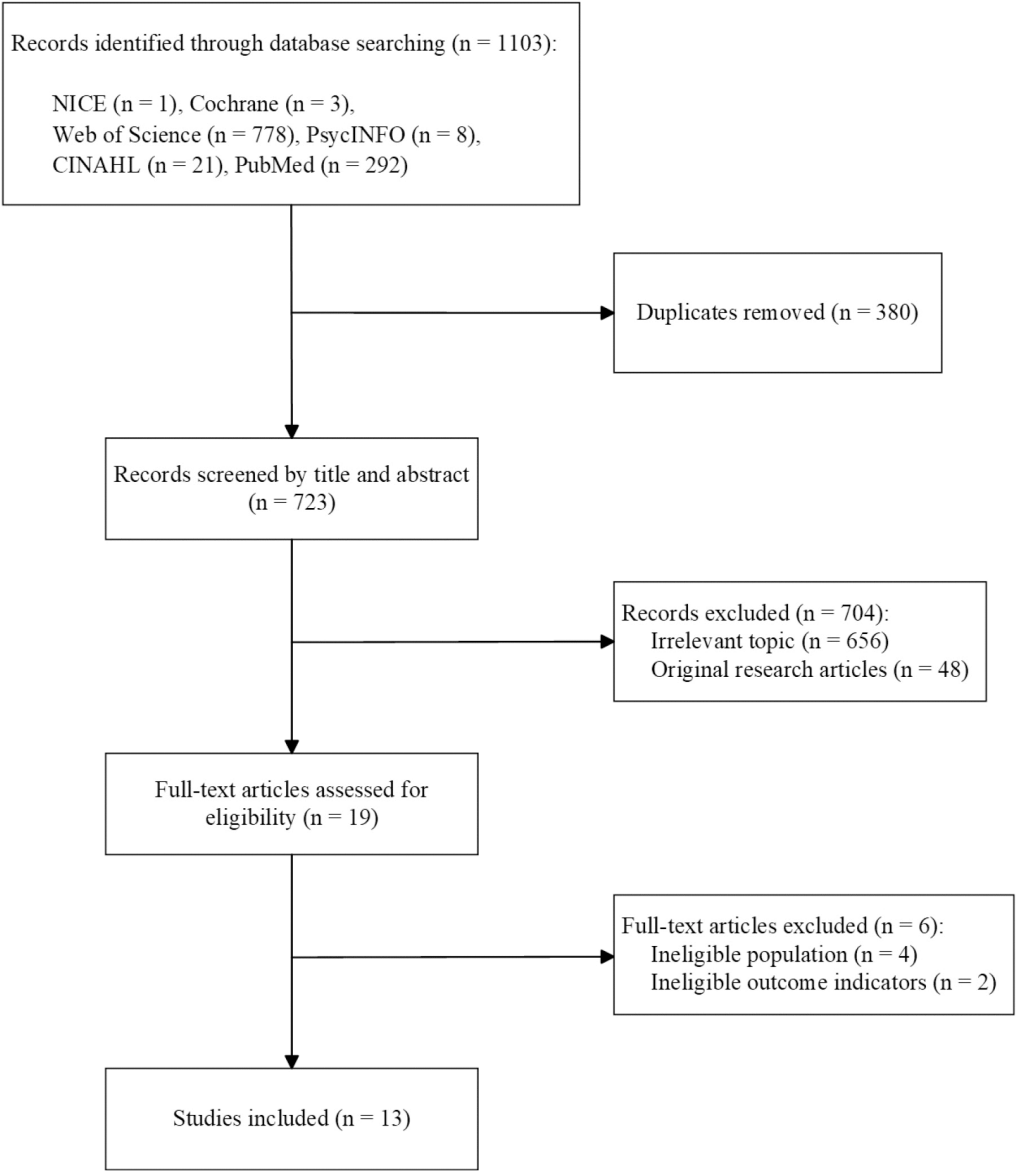
PRISMA flow diagram of literature identification, screening, and inclusion.

The literature search yielded 1,103 records. After removal of 380 duplicates, 723 records remained for screening. Of these, 704 were excluded based on title and abstract screening, and six additional records were excluded after full-text review. Ultimately, one guideline and 12 systematic reviews were included in the final synthesis (Fig 2) [16,17,23–28,32–36].

#### Characteristics of the included evidence.

All included documents were published in English and comprised one guideline and 12 systematic reviews. Their main characteristics are presented in S3 File.

#### Methodological quality of the included evidence.

The included guideline [36] was rated as high quality using AGREE II. The standardized domain scores were 97.0% for Scope and Purpose, 88.9% for Stakeholder Involvement, 94.4% for Rigor of Development, 87.5% for Clarity of Presentation, 75.0% for Applicability, and 100% for Editorial Independence. Inter-rater agreement was 0.747. Although the guideline did not report explicit recommendation grades, it was still considered suitable for informing practice.

All 12 systematic reviews [16,17,23–28,32–35] met the JBI methodological appraisal criteria and were retained for synthesis. Detailed appraisal results are provided in S3 File.

#### Evidence synthesis and thematic categorization.

A total of 64 evidence statements were extracted from the 13 included documents. Through inductive synthesis, these statements were grouped into 12 thematic domains, including caregiver rights, macro-level policy support, multidisciplinary team establishment, intervention formats and settings, and specific intervention approaches. After integration and refinement, 31 best evidence statements were generated and are presented in S3 File.

#### Overview of key evidence themes.

The synthesized evidence highlighted several domains relevant to intervention development. Evidence on caregiver rights emphasized the importance of ensuring caregivers’ access to ASD-related information, support resources, and psychological services, as highlighted in the NICE guideline [36]. Evidence at the policy level underscored the importance of incorporating both children with ASD and their family caregivers into formal mental health support systems to promote family well-being [25]. Evidence on service organization supported multidisciplinary collaboration among healthcare, psychological, and educational professionals to provide comprehensive support for caregivers [16,28].

Regarding intervention delivery, the evidence suggested that group-based formats and implementation in rehabilitation settings may improve feasibility and caregiver engagement [16,28]. Caregiver-focused interventions, which directly address caregivers’ psychological and emotional needs, showed more consistent benefits for caregiver well-being than caregiver-mediated, child-centered approaches [23,28]. Evidence on specific intervention approaches supported the use of mindfulness-based interventions, Acceptance and Commitment Therapy, psychoeducation, problem-solving skills training, expressive writing, and multicomponent programs. Collectively, these approaches were associated with reduced caregiver burden and improved psychosocial outcomes, providing an evidence-informed basis for the preliminary development of the psychosocial intervention program in the present study [16,23–25].

### Phase 2. Preliminary intervention program

The preliminary psychosocial intervention program comprised three core modules, eight intervention themes, and 26 intervention components. The draft program was developed by integrating theory-derived domains with context-specific targets identified from the prior quantitative and qualitative phases and by mapping these targets to evidence-informed intervention strategies. Where multiple sources converged, priority was given to strategies that were both theoretically aligned and practically deliverable within a structured caregiver-focused program.

### Phase 3. Delphi expert consultation

#### Characteristics of Delphi experts.

A total of 16 experts participated in the Delphi consultation. Their mean age was 47.50 ± 4.89 years, and their mean length of professional experience was 23.88 ± 6.11 years. Of the 16 experts, three held bachelor’s degrees, seven held master’s degrees, and six held doctoral degrees. All experts held senior professional titles. Their areas of expertise included autism intervention, psychological therapy, psychiatric nursing, clinical nursing, and nursing education. Detailed characteristics of the experts are presented in Table 3.

**Table 3:** Characteristics of Delphi experts (n = 16).

No.	Sex	Age (years)	Highest degree	Professional title	Years of experience	Research specialty
1	Female	53	PhD	Professor	29	Nursing education
2	Female	55	PhD	Professor	32	Psychiatric mental health nursing
3	Female	45	PhD	Associate professor	22	Psychiatric mental health nursing
4	Female	44	Master’s	Associate professor	17	Nursing education
5	Female	44	Master’s	Associate professor	18	Nursing education
6	Female	46	Bachelor’s	Chief nurse	26	Psychiatric nursing
7	Male	43	Master’s	Associate chief physician	18	Treatment of mental disorders
8	Male	52	Bachelor’s	Chief physician	30	Autism intervention
9	Male	45	Master’s	Associate chief physician	20	Psychotherapy
10	Female	55	Bachelor’s	Chief nurse	35	Psychiatric nursing
11	Female	45	PhD	Associate professor	21	Nursing education; chronic disease nursing
12	Female	46	Master’s	Associate professor	22	Clinical nursing; nursing management
13	Female	53	Master’s	Associate professor	29	Clinical nursing
14	Female	51	Master’s	Professor	29	Clinical nursing; nursing management
15	Female	44	PhD	Associate professor	19	Nursing education
16	Female	39	PhD	Associate professor	15	Nursing education; nursing humanities

#### Expert engagement and authority.

Two rounds of Delphi consultation were completed. In each round, 16 questionnaires were distributed by WeChat or email, and all were returned with valid responses, yielding a response rate of 100%.

Expert authority was assessed using the authority coefficient (Cr). The judgment basis coefficient (Ca) was 0.90 and the familiarity coefficient (Cs) was 0.76, resulting in a Cr of 0.83, which indicated a high level of expert authority. Detailed results on expert engagement and authority are shown in Table 4.

**Table 4:** Expert participation and authority in the Delphi consultation.

Item	Expert response rate	Effective response rate	Expert authority coefficient (Cr)
Value	100%	100%	0.83

#### First-round Delphi results and program revisions.

In the first round, all items met the predefined item-level retention thresholds, with mean importance scores of at least 3.50 and CVs of no more than 0.25. Kendall’s coefficient of concordance was 0.23 (χ² = 132.04, P < 0.001), indicating statistically significant but modest agreement across the multidisciplinary panel. These results supported continued refinement of the item set but were not interpreted as strong consensus.

At the first level, all three core modules including stressors, cognitive appraisal, and coping met the predefined retention criteria and were retained. At the second level, all eight intervention themes were retained. At the third level, all 26 intervention components were also retained based on the quantitative ratings. Detailed first-round ratings are presented in Table 5.

**Table 5:** Results of the first-round Delphi consultation.

Level	Item	Mj	SD	CV	Expert comments
Level 1	Stressors	4.63	0.62	0.13	None
	Cognitive appraisal	5.00	0.00	0.00	Three experts suggested that the intervention content within the cognitive appraisal and coping modules should be more targeted and revised according to the characteristics of the study population.
	Coping	5.00	0.00	0.00	Two experts suggested further improving the overall feasibility of the intervention.
Level 2	Basic knowledge of ASD	4.88	0.34	0.07	One expert suggested delivering the intervention through face-to-face lectures combined with educational videos.
	Sources of stress among FCs	4.94	0.25	0.05	None
	Expectations regarding illness prognosis	4.94	0.25	0.05	Two experts suggested merging this item into “Basic knowledge of ASD.”
	Psychological capital	4.94	0.25	0.05	One expert suggested clarifying whether the distribution of practice videos and e-books was optional and specifying the duration of each mindfulness practice session.
	Perceived social support	4.94	0.25	0.05	One expert suggested revising or removing Intervention Objective (1).
	Illness cognition	4.94	0.25	0.05	One expert suggested improving the specificity of ACT-based intervention content.
	Affiliate stigma	4.94	0.25	0.05	One expert suggested adding reflective journaling to the intervention format.
	Coping styles	4.94	0.25	0.05	None
Level 3	Etiology and pathogenesis	4.19	0.66	0.16	One expert recommended adding content on factors that may aggravate or alleviate ASD-related symptoms.
	Clinical manifestations	4.94	0.25	0.05	One expert recommended further clarifying how the intervention would be evaluated or revising the evaluation content.
	Diagnosis and treatment	4.50	0.63	0.14	Two experts recommended adding key nursing considerations; one expert recommended adding content on educational and training methods for common problem behaviors.
	Caregiving tasks and associated difficulties	4.88	0.34	0.07	Two experts recommended adding an ice-breaking activity to enhance mutual understanding and trust among group members.
	Introduction to prognosis-related knowledge	5.00	0.00	0.00	None.
	Sharing expectations regarding illness prognosis	4.75	0.58	0.12	One expert recommended guiding FCs during the classroom activity to specify their expectations regarding prognosis. FCs could first identify the minimum expected outcome, thereby lowering expectations and enhancing their sense of achievement, and then progressively raise their expectations while explaining the rationale and likelihood, until reaching the best outcome that their child might realistically achieve. Another expert suggested considering whether guidance provided in front of the group when FCs hold unrealistic expectations might elicit negative emotions.
	Overview of mindfulness	4.69	0.60	0.13	One expert recommended replacing “overview” with “introduction” or “knowledge-based instruction.”
	Mindful breathing	4.81	0.54	0.11	None.
	Body scan	4.81	0.54	0.11	None.
	Mindful eating	4.69	0.60	0.13	One expert recommended providing a more detailed description of the “raisin exercise.”
	Mindful stretching	4.69	0.60	0.13	None.
	Mindful sitting	4.69	0.60	0.13	None.
	Emotional awareness	4.88	0.34	0.07	None.
	Mindful walking	4.81	0.54	0.11	One expert suggested increasing the duration allocated to mindful walking.
	Gratitude-based intervention	4.81	0.40	0.08	One expert suggested adding gratitude toward professional support resources in addition to family members; another expert recommended clarifying the practical operability of the intervention and specifying guidance strategies.
	Expressive writing	4.69	0.48	0.10	One expert suggested revising the intervention evaluation approach.
	Introduction to acceptance and commitment therapy	4.69	0.48	0.10	One expert suggested replacing “overview” with “introduction” or “knowledge-based instruction.”
	Acceptance and cognitive defusion training	5.00	0.00	0.00	Two experts suggested adding principles for adjusting training intensity; one expert suggested clarifying how participants are guided to accept themselves.
	Values clarification	4.81	0.40	0.08	None
	Values-based committed action	4.75	0.68	0.14	None
	Advanced training in acceptance and cognitive defusion	4.88	0.34	0.07	One expert suggested that training methods should be more experiential and that this item could be merged with “Acceptance and cognitive defusion training.”
	Self-observation	4.88	0.34	0.07	None
	Introduction to self-compassion	4.94	0.25	0.05	One expert suggested replacing “overview” with “introduction” or “knowledge-based instruction.”
	Self-compassion imagery	4.81	0.40	0.08	None
	Self-compassion dialogue	4.88	0.34	0.07	One expert suggested aligning dialogue content more closely with affiliate stigma.
	Problem-solving skills	5.00	0.00	0.00	None

In addition to the quantitative ratings, experts provided qualitative suggestions to improve the clarity, feasibility, and implementation of the program. Regarding intervention delivery, experts suggested combining online and face-to-face formats, providing recorded sessions for caregivers unable to attend in person, limiting group size to facilitate interaction, and adjusting the frequency of check-in activities to accommodate caregivers’ time constraints. Regarding program structure, experts recommended reviewing key content from the previous session at the beginning of each session, specifying the intervention provider for each component, and distinguishing between whole-group and small-group discussion formats. Regarding evaluation and outcome assessment, experts suggested aligning intervention objectives with corresponding evaluation indicators, clarifying evaluation criteria, and specifying assessment time points. In addition, several experts recommended clarifying whether post-intervention support would be provided through the online communication group and developing contingency plans for unexpected situations or participant non-adherence during implementation. All qualitative comments were reviewed and discussed by the research team and informed subsequent program refinement.

Based on the first-round results and research team discussions, the preliminary intervention program was revised to improve conceptual clarity, feasibility, and internal coherence while preserving all core intervention targets. Revisions mainly involved merging conceptually overlapping items, refining terminology and session focus, and streamlining components with substantial content overlap. For example, content related to ASD etiology, clinical manifestations, and treatment was integrated into one component on basic ASD-related knowledge within the stressor module. Introductory content related to mindfulness, Acceptance and Commitment Therapy, and self-compassion was reframed as intervention introduction content rather than theoretical overview content. In addition, several components were merged or removed when expert feedback indicated redundancy or limited added value within the available session time. For example, mindfulness meditation was integrated with mindfulness breathing exercises, and advanced acceptance and cognitive defusion content was merged to streamline program delivery. These revisions were made on the basis of feasibility considerations and expert recommendations rather than statistical exclusion, and no core intervention target was removed. After revision, the program comprised three core modules, seven intervention themes, and 22 intervention components.

#### Second-round Delphi results and program finalization.

In the second round, all retained items continued to meet the predefined item-level criteria, with mean importance scores ≥3.50 and CVs ≤ 0.25. Kendall’s W increased only marginally from 0.23 to 0.25 and remained modest. The statistically significant coefficient therefore indicated non-random concordance, but not strong agreement among experts.

All three core modules, seven themes, and 22 components met the predefined item-level criteria and were retained after review of their ratings and written comments. Retention reflects satisfaction of the prespecified decision rules, not strong unanimity across the panel (Table 6).

**Table 6:** Results of the second-round Delphi consultation.

Level	Item	Mj	SD	CV	Expert comments
Level 1	Stressors	4.86	0.34	0.07	None
	Cognitive appraisal	5.00	0.00	0.00	One expert suggested placing *perceived social support* at the same level as *cognitive appraisal* and listing it as a separate item.
	Coping	5.00	0.00	0.00	None
Level 2	Sources of stress among FCs	4.75	0.45	0.09	One expert suggested revising the intervention objective to include “acquisition of knowledge”; one expert suggested modifying the evaluation method so that each participant can articulate the process through which stress is generated.
	Expectations regarding illness prognosis	4.81	0.40	0.08	One expert suggested changing the evaluation method to a group representative presentation demonstrating that group members are able to form realistic expectations; one expert suggested introducing key practice points before the exercise.
	Psychological capital	5.00	0.00	0.00	One expert suggested revising Intervention Objective (1) to include “recognition and acceptance of mindfulness-based therapy”; one expert suggested changing the intervention format from “demonstration” to “guided demonstration.”
	Perceived social support	5.00	0.00	0.00	None
	Illness cognition	5.00	0.00	0.00	One expert suggested revising Intervention Objective (1) to emphasize that suffering is inevitable and that FCs should learn to live a meaningful life despite suffering; one expert suggested incorporating emotional acceptance alongside cognitive defusion during the warm-up and review sessions; one expert suggested changing the intervention format from “demonstration” to “guided demonstration.”
	Affiliate stigma	5.00	0.00	0.00	One expert suggested that intervention objectives should focus on affiliate stigma; one expert suggested changing the intervention format from “demonstration” to “guided demonstration.”
	Coping styles	5.00	0.00	0.00	One expert suggested adding role-play to the intervention format.
Level 3	Psychological processes through which stressors affect individuals	4.81	0.40	0.08	None
	Basic knowledge of ASD	4.75	0.45	0.09	None
	Introduction to knowledge of illness prognosis	4.69	0.48	0.10	None
	Clarifying FCs’ expectations regarding illness prognosis	5.00	0.00	0.00	None
	Introduction to mindfulness-based intervention	4.69	0.48	0.10	One expert suggested elaborating on the effects of mindfulness-based intervention.
	Mindful breathing	4.81	0.40	0.08	None
	Body scan	4.75	0.45	0.09	None
	Mindful eating	5.00	0.00	0.00	None
	Mindful stretching	5.00	0.00	0.00	None
	Emotional awareness	4.56	0.51	0.11	Two experts suggested encouraging FCs to reflect on recent emotional fluctuations or their emotional states at the time of the child’s diagnosis.
	Mindful walking	5.00	0.00	0.00	None
	Gratitude-based intervention	5.00	0.00	0.00	One expert suggested adding specific practices, such as “three good things.”
	Expressive writing	5.00	0.00	0.00	One expert suggested providing a structured writing framework.
	Introduction to acceptance and commitment therapy	4.69	0.48	0.10	None
	Acceptance and cognitive defusion training	5.00	0.00	0.00	One expert suggested clarifying the distinction between emotional acceptance and cognitive defusion, as these concepts are easily confused.
	Self-observation	5.00	0.00	0.00	None
	Values clarification	4.75	0.45	0.09	None
	Values-based committed action	4.50	0.52	0.11	None
	Introduction to self-compassion	5.00	0.00	0.00	None
	Self-compassion meditation	5.00	0.00	0.00	None
	Self-compassion dialogue	5.00	0.00	0.00	One expert suggested setting specific dialogue prompts.
	Problem-solving skills	5.00	0.00	0.00	None

In addition to quantitative ratings, experts provided qualitative feedback during the second round of consultation, primarily focusing on further refinement of intervention delivery, clarity, and risk management. Suggestions related to intervention format included replacing the term “self-introduction” with “ice-breaking activity,” clearly distinguishing between whole-group discussion and small-group discussion, adjusting session duration, and specifying acceptable formats for homework submission (e.g., video, photographs, written text, or audio recordings). Experts also recommended refining the names of intervention modules and themes and providing more detailed descriptions of video-based materials used during the intervention to enhance clarity and consistency. With respect to evaluation and safety considerations, experts suggested elaborating on intervention evaluation methods and developing an emergency response plan to address potential situations of marked emotional distress among participants. All qualitative suggestions were reviewed and discussed by the research team and informed further refinement of the intervention program.

Based on the second-round ratings, written feedback, and subsequent research-team discussion, the overall structure was retained while content-level refinements were made. Intervention objectives were sharpened; selected exercises and homework activities were specified; and supporting materials, including basic ASD information, structured writing templates, and interview or dialogue guides, were added to support standardized delivery. After two rounds, the finalized program comprised three core modules, seven themes, and 22 components. A concise summary of the finalized program is provided in Table 7 to enhance transparency and replicability, whereas detailed session objectives, activities, and supporting materials are presented in S4 File.

**Table 7:** Summary of the finalized psychosocial intervention program for family caregivers of children with ASD.

Session	Module	Theme	Key content	Format	Duration	Provider	Evaluation method
**1**	Stressors/Cognitive appraisal	1.1 Sources of stress among FCs 2.1 Expectations regarding illness prognosis	Stress pathways and mechanisms; basic ASD knowledge; introduction to illness prognosis; sharing and clarifying expectations	Lecture (online with recorded playback); group discussion	90–120 min	Researchers, pediatricians, psychiatrists	Knowledge assessment; group representative presentation
**2**	Cognitive appraisal	2.2 Psychological capital	Introduction to mindfulness; mindful breathing; body scan; mindful eating; case sharing	Lecture; guided demonstration; in-session practice	90–120 min	Researchers, psychologists, nurses	Assessment of understanding and application of mindfulness techniques
**3**	Cognitive appraisal	2.2 Psychological capital (continued)	Mindful stretching; emotional awareness; mindful walking; integration practice	Sharing; guided demonstration; in-session practice	90–120 min	Researchers, psychologists, nurses	Assessment of mindfulness application and enhancing psychological capital
**4**	Cognitive appraisal	2.3 Perceived social support	Gratitude-based intervention; expressive writing; three good things exercise	Sharing; discussion; lecture; in-session practice	90–120 min	Researchers	Whether FCs can demonstrate specific supportive behaviors
**5**	Cognitive appraisal	2.4 Illness cognition	Introduction to ACT; emotional acceptance; cognitive defusion training; self-observation	Sharing; discussion; lecture; guided demonstration; practice	90–120 min	Researchers, psychologists, nurses	Based on completion of ACT exercises and discussion outcomes
**6**	Cognitive appraisal	2.4 Illness cognition (continued)	Values clarification; values-based committed actio	Sharing; discussion; lecture; practice	90–120 min	Researchers, psychologists, nurses	Classroom discussion and completion of values-based exercises
**7**	Cognitive appraisal	2.5 Affiliate stigma	Introduction to self-compassion; self-compassion meditation; self-compassion dialogue	Sharing; discussion; lecture; guided demonstration	90–120 min	Researchers	Group representative presentation and sharing
**8**	Coping	3.1 Coping strategies	Problem-solving skills training (IDEAL strategy); case-based practice; program summary	Lecture; discussion; sharing; role-play	90–120 min	Researchers, rehabilitation therapists	Each group submits a case report; post-intervention assessment

Implementation planning incorporated several safeguards to reduce burden and support fidelity during subsequent testing. The eight weekly sessions were retained because the content spans distinct informational, appraisal, and coping targets and because the evidence synthesis supported multicomponent programs delivered over approximately 5–8 weeks. Planned burden-reduction measures include hybrid attendance, recorded review materials, small groups, scheduling alongside the child’s rehabilitation where possible, brief breaks, flexible homework formats, and follow-up after missed sessions. Planned fidelity procedures include a standardized manual, facilitator training and rehearsal, session checklists, attendance and homework records, documentation of adaptations and deviations, and regular supervision. Facilitators will monitor fatigue and distress during each session; activities will be paused or shortened when needed, and marked distress will trigger immediate support and referral by a qualified mental-health professional. These procedures are implementation plans, not evidence that the program is already feasible.

## Discussion

Family caregivers of children with ASD frequently experience substantial and multidimensional caregiver burden, which may adversely affect caregivers’ well-being, family functioning, and the child’s rehabilitation and developmental support [9,10]. Although caregiver burden has been widely recognized, existing interventions for this population have often focused on isolated psychological outcomes, such as stress, anxiety, or depressive symptoms, or have been embedded within caregiver-mediated programs primarily targeting child outcomes [16,17,23]. Against this background, the present study aimed to develop a structured psychosocial intervention program specifically designed to target caregiver burden among family caregivers of children with ASD.

A major strength of the present study lies in its theory-driven and evidence-informed development process. The integrated ABC-X family stress model and stress and coping theory organized the intervention around family caregiving stressors, appraisal, resources, and coping. The prior quantitative phase identified potentially modifiable psychosocial targets through a multicentre survey and pathway analyses, while the qualitative phase provided caregiver-derived accounts of lived burden, prognosis expectations, appraisal and coping patterns, and preferences for intervention delivery. These empirical inputs were considered most relevant where they converged with the theoretical framework and best evidence synthesis. Nevertheless, the cross-sectional design of the quantitative phase limits causal inference, and the qualitative findings reflect a small, service-engaged sample; the earlier phases therefore informed target selection rather than independently proving that the selected components will be effective.

The final program addresses the heterogeneity and fragmentation described in previous reviews by organizing multiple caregiver-focused strategies within one explicit framework [23,24,26]. It integrates psychoeducation, mindfulness-based approaches, gratitude-based expressive writing, Acceptance and Commitment Therapy-informed strategies, self-compassion training, and problem-solving skills. Reviews suggest that caregiver-focused and multicomponent approaches may improve psychosocial outcomes, but heterogeneity in content, delivery, and outcomes precludes assuming that the present combination is superior before direct testing [23,24]. The program should therefore be viewed as a structured synthesis of plausible components rather than an established effective package.

The systematic evidence synthesis, transparent item-level decision criteria, high expert participation, and multidisciplinary review enabled systematic development, expert appraisal, and refinement of the program. However, Kendall’s W remained modest in both rounds. The ratings reflect experts’ perceptions of the relevance, clarity, and appropriateness of the program content; they do not constitute evidence of formal content validity, strong consensus, practical feasibility, or effectiveness.

The program provides a structured framework that nurses and other professionals may use in later feasibility testing of caregiver-focused support. Its planned hybrid delivery, small-group activities, standardized materials, and multidisciplinary facilitation were selected to reduce access barriers and support consistent implementation. Nevertheless, eight sessions of 90–120 minutes may be demanding for caregivers who already face substantial time and emotional constraints. Flexible attendance, breaks, recorded materials, follow-up after missed sessions, fatigue monitoring, and distress-management procedures are intended to mitigate this burden. Facilitator training, manuals, session checklists, supervision, and documentation of adaptations are planned to support fidelity. Whether these measures are acceptable and sufficient must be assessed directly with caregivers using recruitment, attendance, retention, completion, adverse-event, fidelity, and qualitative acceptability indicators.

This study has several limitations. First, the program has not yet completed empirical feasibility or effectiveness evaluation. Pilot testing has begun and 12 family caregivers had enrolled at the time of manuscript preparation, but no preliminary process, feasibility, or outcome data are included in the present report. Second, caregivers contributed indirectly through the prior qualitative phase but did not directly co-design or validate the final post-Delphi program. Focus groups or cognitive interviews with caregivers are therefore needed to assess acceptability, cultural fit, burden, emotional safety, and practical feasibility before a definitive trial. Third, the consensus exercise was a two-round asynchronous modified Delphi rather than a synchronous deliberative process. Delphi designs vary and no universal number of rounds is required [37], but the format limited real-time clarification and may have contributed to the modest W values. Fourth, the panel was small and recruited within one national context, and the evidence synthesis included one guideline and 12 systematic reviews with heterogeneous underlying interventions. Finally, the eight-session multicomponent program may create participation burden and implementation complexity; the adequacy of the planned flexibility and fidelity safeguards remains to be established.

## Conclusion

Guided by an integrated theoretical framework and informed by prior quantitative and qualitative findings, best evidence synthesis, and a modified Delphi consultation, this study developed a structured psychosocial intervention framework for caregiver burden among family caregivers of children with ASD. The final program comprised three core modules, seven themes, and 22 components. The Delphi process enabled systematic refinement and expert appraisal of the program but did not establish formal content validity, feasibility, or effectiveness. The framework is ready for direct caregiver-informed feasibility testing, including assessment of acceptability, delivery fidelity, participant burden, cultural fit, and emotional safety, before subsequent effectiveness evaluation.

## Supporting information

S1 File Literature search. (DOCX)

S2 File Basis for calculating expert authority. (DOCX)

S3 File Results of best evidence synthesis. (DOCX)

S4 File Finalized psychosocial intervention program for caregiver burden among family caregivers of children with ASD. (DOCX)
