# Supplementary material for: An evidence-informed psychosocial intervention program for caregiver burden among family caregivers of children with autism spectrum disorder: a multi-stage development and Delphi study
Source: PLoS One. 2026 Aug 12;21(8):e0354823. doi: 10.1371/journal.pone.0354823 (PMC13465810; doi:10.1371/journal.pone.0354823)
Supplement: Supplementary file 1 [file pone.0354823.s005.pdf]

Review Report: PONE-D-26-16776

The authors propose a multidisciplinary intervention program that will contribute significantly to both the Sustainable Development Goals (SDGs: 3, 4, 5) and the fields of health and behavioral sciences. A strength of the study is the evaluation of the quality of evidence and the components of the intervention program.

1. Statements of interest and the authors' position on the knowledge gap

Introduction

The authors justify the need to develop a multidisciplinary intervention based on a Rapid Review, three scientific theories, and expert consultation. They also highlight the need to adopt an intervention that goes beyond merely reducing symptoms, opting instead for a structured, multi-component, multidisciplinary psychosocial intervention, as recommended by the WHO (World Health Organization). They also discuss the epidemiology of ASD (Autism Spectrum Disorder) in children, though without providing global figures to support their claims. Therefore, it is appropriate for the authors to note the following:

a) The program's specific contribution, in addition to medical sciences, to clinical and health psychology, and to developmental psychology (because the program will improve the three-way interaction between caregivers, children, and family members).

b) The updated global epidemiology regarding the workload of caregivers of children with ASD. In this regard, it is known that approximately one in every 127 children has ASD, and between 23–27% of these children's caregivers experience psychological health problems with a workload of up to 13–16 hours per day.

Highlight the advantages of using the scientific theories presented in Figure 1 compared to the "Karasek Theoretical Model of Demand-Control-Psychosocial Support in the Work Environment," an analysis of the theoretical perspective of which can be found on pages 8 and 9 of:

https://www.medwave.cl/medios//research/studies/2986/medwave_2024_2986-1.pdf

2. Details of the methods and techniques used

Methods

They are sufficient. However, after review, it should be noted that the study is based on the ACCORD (ACcurate COnsensus Reporting Document) guideline from the EQUATOR Network published by Gattrell et al. (2024) in PLOS Med, 21.

Literature search: the end date is indicated, but the start date is missing.

At the end of the paragraph "Phase 2. Theoretical basis… insert (Figure 1).

2. Data support and analysis techniques

Sufficient.

3. Data availability

In addition to sufficient data availability as supplementary material and deposited in "Zenodo," the evidence synthesis is also registered (ES20245306).

4. Ethical considerations

Adequate. The research project was approved by a Research Ethics Committee (KY-2024-368).

Discussion

The authors state "...the most fragmented intervention approaches when combining multiple components within a single structured framework..."; please cite the references supporting this claim (2nd paragraph).

As limitations:

a) Note that the Delphi method was conducted in a single phase consisting of two rounds of asynchronous consultations (the complete Delphi method comprises three phases with three rounds of in-person or synchronous consultations in the second phase, i.e., controlled and with feedback (Shang, 2023: https://doi.org/10. 1097/MD.0000000000032829).

b) Ideally, the intervention program should be validated through focus groups (caregivers of children with ASD).
