# Supplementary material for: An evidence-informed psychosocial intervention program for caregiver burden among family caregivers of children with autism spectrum disorder: a multi-stage development and Delphi study
Source: PLoS One. 2026 Aug 12;21(8):e0354823. doi: 10.1371/journal.pone.0354823 (PMC13465810; doi:10.1371/journal.pone.0354823)
Supplement: Supplementary file 2 [file pone.0354823.s001.docx]

**S1 File. Literature search**

**1. Databases and information sources**

The literature search was conducted following the 6S evidence hierarchy, with evidence sources searched in a top–down manner to identify the best available evidence relevant to caregiver burden interventions among family caregivers of children with autism spectrum disorder (ASD).

Evidence-based clinical knowledge resources included UpToDate and BMJ Best Practice. Guideline repositories comprised the Guidelines International Network (GIN), the National Institute for Health and Care Excellence (NICE), the Scottish Intercollegiate Guidelines Network (SIGN), the National Guideline Clearinghouse (NGC), the Registered Nurses’ Association of Ontario (RNAO), and the Medlive guideline platform.

Evidence summary resources were searched using the JBI CONNECT^+^ Evidence Summary Database. Databases for systematic reviews included the Cochrane Database of Systematic Reviews (CDSR) and the Joanna Briggs Institute (JBI) Database of Systematic Reviews and Implementation Reports.

In addition, comprehensive bibliographic databases were searched, including Web of Science, PsycINFO, CINAHL, and PubMed, as well as major Chinese databases: China National Knowledge Infrastructure (CNKI), Wanfang Data, VIP Database, and the Chinese Biomedical Literature Database (CBM).

All databases were searched from inception to 15 September 2024.

**2. Search terms**

Search terms were developed using a combination of controlled vocabulary (where applicable) and free-text keywords. The English search terms included:

*“Autism Spectrum Disorder” OR “ASD” AND caregiver* OR parent* AND burden OR stress AND therapy OR intervention AND psychological capital* OR perceived social support OR illness perception OR disease cognition OR affiliate stigma OR psychological preparation OR expectation management OR coping strategy AND guideline OR evidence* OR systematic review OR meta* OR recommendation* OR best practice* OR consensus.*

For Chinese databases, the following terms were used:

*ASD, 孤独症谱系障碍, 照顾者, 父母, 家长, 负担, 压力, 疗法, 治疗, 心理资本, 领悟社会支持, 疾病认知, 连带病耻感, 预期管理, 应对方式, 指南, 证据总结, 最佳实践, 临床决策, 系统评价, 专家共识, Meta 分析.*

**3. Search strategy** **(Excerpt from English)**

Web of Science:

TS=("Autism Spectrum Disorder" OR "ASD" OR "Autism Spectrum Disorders" OR "Autism" OR "Autistic Disorder" OR "Autistic Disorders") AND TS=("caregivers" OR "caregiver" OR "carers" OR "carer" OR "family member*" OR "relatives" OR "relative" OR "mother" OR "father" OR "parent") AND TS=("therapy" OR "intervention" OR "psychosocial" OR "treatment" OR "support" OR "approach" OR "strategies" OR "psychological capitals" OR "psychological capital" OR "perceived social support" OR "illness perception" OR "disease perception" OR "illness cognition" OR "disease cognition" OR "perception of disease" OR "perception of illness" OR "affiliate stigma" OR "stigma" OR "psychological preparation" OR "psychological readiness" OR "psychology readiness" OR "coping strategy" OR "coping style") AND TS=("guideline" OR "evidence*" OR "systematic review" OR "meta" OR "recommendation*" OR "best practice*" OR "consensus")

PsycINFO:

SU ( "Autism Spectrum Disorder" OR ASD OR "Autism Spectrum Disorders" OR Autism OR "Autistic Disorder" OR "Autistic Disorders" OR Asperger* ) AND SU ( caregivers OR caregiver OR carers OR carer OR "family member*" OR mother OR father OR maternal OR paternal OR parent* ) AND SU ( "caregiver burden" OR burden OR stress OR strain OR fatigue OR exhaustion OR burnout ) AND SU ( therapy OR intervention OR psychosocial OR treatment OR support OR approach OR strategies OR "psychological capitals" OR "psychological capital" OR "perceived social support" OR "understanding social support" OR "illness perception" OR "disease perception" OR "illness cognition" OR "disease cognition" OR "perception of disease" OR "perception of illness" OR "affiliate stigma" OR stigma OR "psychology preparing" OR "psychological preparation" OR "psychological readiness" OR "psychology readiness" OR "coping strategy" OR "coping style" ) AND SU ( guideline OR evidence* OR "systematic review" OR meta OR recommendation* OR "best practice*" OR consensus )

CINAHL:

SU ( "Autism Spectrum Disorder" OR ASD OR "Autism Spectrum Disorders" OR Autism OR "Autistic Disorder" OR "Autistic Disorders" OR Asperger* ) AND SU ( caregivers OR caregiver OR carers OR carer OR "family member*" OR mother OR father OR maternal OR paternal OR parent* ) AND SU ( "caregiver burden" OR burden OR stress OR strain OR fatigue OR exhaustion OR burnout ) AND SU ( therapy OR intervention OR psychosocial OR treatment OR support OR approach OR strategies OR "psychological capitals" OR "psychological capital" OR "perceived social support" OR "understanding social support" OR "illness perception" OR "disease perception" OR "illness cognition" OR "disease cognition" OR "perception of disease" OR "perception of illness" OR "affiliate stigma" OR stigma OR "psychology preparing" OR "psychological preparation" OR "psychological readiness" OR "psychology readiness" OR "coping strategy" OR "coping style" ) AND SU ( guideline OR evidence* OR "systematic review" OR meta OR recommendation* OR "best practice*" OR consensus )

PubMed:

("Autism Spectrum Disorder"[Title/Abstract] OR "ASD"[Title/Abstract] OR "Autism Spectrum Disorders"[Title/Abstract] OR "Autism"[Title/Abstract] OR "Autistic Disorder"[Title/Abstract] OR "Autistic Disorders"[Title/Abstract] OR "asperger*"[Title/Abstract]) AND ("caregivers"[Title/Abstract] OR "caregiver"[Title/Abstract] OR "carers"[Title/Abstract] OR "carer"[Title/Abstract] OR "family member*"[Title/Abstract] OR "mother"[Title/Abstract] OR "father"[Title/Abstract] OR "maternal"[Title/Abstract] OR "paternal"[Title/Abstract] OR "parent*"[Title/Abstract]) AND ("caregiver burden"[Title/Abstract] OR "burden"[Title/Abstract] OR "stress"[Title/Abstract] OR "strain"[Title/Abstract] OR "fatigue"[Title/Abstract] OR "exhaustion"[Title/Abstract] OR "burnout"[Title/Abstract]) AND ("therapy"[Title/Abstract] OR "intervention"[Title/Abstract] OR "psychosocial"[Title/Abstract] OR "treatment"[Title/Abstract] OR "support"[Title/Abstract] OR "approach"[Title/Abstract] OR "strategies"[Title/Abstract] OR "psychological capitals"[Title/Abstract] OR "psychological capital"[Title/Abstract] OR "perceived social support"[Title/Abstract] OR "illness perception"[Title/Abstract] OR "disease perception"[Title/Abstract] OR "illness cognition"[Title/Abstract] OR "disease cognition"[Title/Abstract] OR "perception of disease"[Title/Abstract] OR "perception of illness"[Title/Abstract] OR "affiliate stigma"[Title/Abstract] OR "stigma"[Title/Abstract] OR "psychological preparation"[Title/Abstract] OR "psychological readiness"[Title/Abstract] OR "psychology readiness"[Title/Abstract] OR "coping strategy"[Title/Abstract] OR "coping style"[Title/Abstract]) AND ("guideline"[Title/Abstract] OR "evidence*"[Title/Abstract] OR "systematic review"[Title/Abstract] OR "meta"[Title/Abstract] OR "recommendation*"[Title/Abstract] OR "best practice*"[Title/Abstract] OR "consensus"[Title/Abstract]
