# Supplementary material for: An evidence-informed psychosocial intervention program for caregiver burden among family caregivers of children with autism spectrum disorder: a multi-stage development and Delphi study
Source: PLoS One. 2026 Aug 12;21(8):e0354823. doi: 10.1371/journal.pone.0354823 (PMC13465810; doi:10.1371/journal.pone.0354823)
Supplement: Supplementary file 3 [file pone.0354823.s002.docx]

**S2 File. Basis for calculating expert authority**

**Table S1 Weighting scheme for experts’ judgment basis**

| **Judgment basis** | **High impact** | **Moderate impact** | **Low impact** |
| --- | --- | --- | --- |
| Practical experience | 0.5 | 0.4 | 0.3 |
| Theoretical analysis | 0.3 | 0.2 | 0.1 |
| Reference to domestic and international literature | 0.1 | 0.1 | 0.1 |
| Intuitive judgment | 0.1 | 0.1 | 0.1 |

**Table S2. Scoring criteria for experts’ familiarity with the consultation content**

| **Level of familiarity** | **Very familiar** | **Familiar** | **Moderate** | **Somewhat familiar** | **Unfamiliar** |
| --- | --- | --- | --- | --- | --- |
| Score | 1.0 | 0.8 | 0.5 | 0.2 | 0.0 |
