# Supplementary material for: An evidence-informed psychosocial intervention program for caregiver burden among family caregivers of children with autism spectrum disorder: a multi-stage development and Delphi study
Source: PLoS One. 2026 Aug 12;21(8):e0354823. doi: 10.1371/journal.pone.0354823 (PMC13465810; doi:10.1371/journal.pone.0354823)
Supplement: Supplementary file 4 [file pone.0354823.s003.docx]

**S3 File. Results of best evidence synthesis**

**Table S3. Characteristics of the included studies**

| **No.** | **Author** | **Year** | **Type of evidence** | **Main content** |
| --- | --- | --- | --- | --- |
| 1 | NICE [1] | 2011 | Evidence-based guideline | Provides individualized information support for families of children with ASD and their family caregivers (FCs), including local and national support organizations (which offer opportunities for families to communicate with others with similar experiences or provide information on specific courses), welfare benefits, educational support, and social care information, as well as preparation information for the child’s transition to adulthood. These resources help ASD families cope with current and future challenges. |
| 2 | Yesilkaya et al. [2] | 2024 | Systematic review | Examines the relationship between ASD severity in children and parental stress, and evaluates the effectiveness of mindfulness interventions in reducing parental stress. Included studies comprised randomized controlled trials (RCTs), quasi-experimental studies, pre–post studies, and observational studies. The authors found a positive association between parental stress levels and ASD severity. Mindfulness interventions were shown to have beneficial effects in reducing parental stress; however, further research is needed to more comprehensively understand the impact of these interventions. |
| 3 | Li, S. N. et al. [3] | 2024 | Systematic review and meta-analysis | Evaluates the effectiveness of parent-focused interventions for parents of children with ASD. Included studies were RCTs, and parent-focused interventions comprised cognitive behavioral therapy, mindfulness, acceptance and commitment therapy (ACT), integrated interventions, psychoeducation programs, and expressive writing. Following intervention, parental stress, depressive symptoms, and distress, as well as children’s problem behaviors, were reduced, and parent–child relationships were improved. The certainty of evidence ranged from moderate to high. |
| 4 | Agarwal et al. [4] | 2024 | Systematic review | Examines interventions targeting stress and anxiety among parents of transition-aged children with ASD. Included studies comprised randomized controlled trials (RCTs), quasi-experimental studies, and pre–post studies. Intervention approaches included mindfulness training, social skills interventions, and multi-component interventions. Although these interventions demonstrated some effectiveness in reducing parental stress and anxiety, substantial heterogeneity was observed across studies, with variability in intervention effects and sustainability. |
| 5 | Ağırkan et al. [5] | 2023 | Systematic review and meta-analysis | Evaluates the effectiveness of group-based psychoeducation programs for parents of children with ASD. Included studies comprised RCTs and quasi-experimental studies. Group-based psychoeducation programs included ASD-related information and education, parenting skills training, and parental stress management strategies. Following intervention, parents’ psychological symptoms and well-being improved, and the type and frequency of psychoeducation programs had significant moderating effects on psychological outcomes. |
| 6 | MacKenzie et al. [6] | 2022 | Systematic review and meta-analysis | Assesses the effects of parent-focused interventions on parents of children with ASD. Included studies were RCTs, and interventions comprised psychoeducation programs and parent-mediated interventions targeting children’s core symptoms and problem behaviors. These interventions produced small but significant improvements in parental self-efficacy and, to some extent, psychological health; however, no significant improvements were observed in caregiver burden, family adaptation, or physical health. |
| 7 | Kulasinghe et al. [7] | 2022 | Systematic review and meta-analysis | Evaluates the effects of psychological interventions on the mental health and parent–child relationships of parents of children with ASD. Included studies were RCTs, and interventions comprised psychoeducation programs and parent-mediated interventions. Responsive parenting strategies had positive effects on parent–child relationships and reduced parenting stress, whereas other psychoeducation programs and parent-mediated interventions showed no significant effects on parental depressive symptoms or general psychological health. |
| 8 | Juvin et al. [8] | 2022 | Systematic review | Evaluates the effectiveness of acceptance and commitment therapy (ACT) for parents of children with ASD. Included studies comprised RCTs and quasi-experimental studies. ACT demonstrated beneficial effects on parents’ psychological health and quality of life. |
| 9 | Hartley et al. [9] | 2022 | Meta-synthesis | Synthesizes barriers and facilitators related to mindfulness- and acceptance and commitment therapy (ACT)–based interventions for children with ASD and their caregivers. Included studies comprised quantitative, qualitative, and mixed-methods research. Interventions primarily involved mindfulness and ACT. These interventions were found to enhance individuals’ self-awareness, self-regulation, and self-compassion, thereby reducing distress and benefiting families. |
| 10 | Merriman et al. [10] | 2020 | Systematic review | Evaluates the effects of clinician-led, parent-focused interventions on parental outcomes. Included studies comprised experimental studies, quasi-experimental studies, and mixed-methods research. Interventions included mindfulness, cognitive behavioral therapy, ACT, and non-directive counseling. Parent-focused interventions demonstrated positive effects on parents’ psychological health and well-being and may ultimately benefit all family members. |
| 11 | Yu et al. [11] | 2019 | Systematic review and meta-analysis | Examines the effects of family caregiver (FC)-focused interventions on improving psychosocial outcomes among FCs of children with ASD. Included studies comprised RCTs and quasi-experimental studies. Interventions included psychoeducation, mindfulness/ACT, social support, expressive writing, and multi-component interventions. FC-focused interventions showed positive effects on overall psychosocial outcomes, with mindfulness/ACT demonstrating greater effectiveness than other approaches. |
| 12 | Rutherford et al. [12] | 2019 | Systematic review and meta-analysis | Investigates interventions for parents of children with ASD aged 7 years and older and their effects on parental physical and mental health. Included studies comprised RCTs, quasi-experimental studies, and pre–post studies. Interventions included parent education, mindfulness or relaxation training, parent support groups, and multi-component parent–child intervention programs. Parent education and mindfulness training demonstrated significant effects in reducing parental stress, improving parenting styles, and increasing satisfaction. |
| 13 | Catalano et al. [13] | 2018 | Systematic review | Evaluates the effectiveness of mental health interventions for parents caring for children with ASD. Included studies comprised RCTs, quasi-experimental studies, and mixed-methods research. Interventions included psychoeducation programs, mindfulness, ACT, and online support groups. These interventions improved parents’ psychological mental health and contributed to enhanced health-related quality of life in families of children with ASD. |

**Table S4. AGREE II quality appraisal of the included guideline**

| **Domain** | **Item** | **Reviewer 1** | **Reviewer 2** | **Score (%)** |
| --- | --- | --- | --- | --- |
| Scope and purpose | 1. The overall objective(s) of the guideline is (are) specifically described | 7 | 7 | 97.0 |
|  | 2. The health question(s) covered by the guideline is (are) specifically described | 7 | 6 |  |
|  | 3. The population (patients, public, etc.) to whom the guideline is meant to apply is specifically described | 7 | 7 |  |
| Stakeholder involvement | 4. The guideline development group includes individuals from all relevant professional groups | 6 | 7 | 88.9 |
|  | 5. The views and preferences of the target population (patients, public, etc.) have been sought | 5 | 6 |  |
|  | 6. The target users of the guideline are clearly defined | 7 | 7 |  |
| Rigor of development | 7. Systematic methods were used to search for evidence | 7 | 7 | 94.4 |
|  | 8. The criteria for selecting the evidence are clearly described | 7 | 6 |  |
|  | 9. The strengths and limitations of the body of evidence are clearly described | 6 | 5 |  |
|  | 10. The methods for formulating the recommendations are clearly described | 7 | 6 |  |
|  | 11. The health benefits, side effects, and risks have been considered in formulating the recommendations | 6 | 7 |  |
|  | 12. There is an explicit link between the recommendations and the supporting evidence | 7 | 7 |  |
|  | 13. The guideline has been externally reviewed by experts prior to publication | 7 | 7 |  |
|  | 14. A procedure for updating the guideline is provided | 7 | 7 |  |
| Clarity of presentation | 15. The recommendations are specific and unambiguous | 7 | 7 | 87.5 |
|  | 16. The different options for management of the condition or health issue are clearly presented | 7 | 6 |  |
|  | 17. Key recommendations are easily identifiable | 7 | 7 |  |
| Applicability | 18. The guideline describes facilitators and barriers to its application | 6 | 6 | 75.0 |
|  | 19. The guideline provides advice and/or tools on how the recommendations can be put into practice | 6 | 5 |  |
|  | 20. The potential resource implications of applying the recommendations have been considered | 5 | 5 |  |
|  | 21. The guideline presents monitoring and/or auditing criteria | 6 | 6 |  |
| Editorial independence | 22. The views of the funding body have not influenced the content of the guideline | 7 | 7 | 100 |
|  | 23. Competing interests of guideline development group members have been recorded and addressed | 7 | 7 |  |

**Table S5 JBI methodological quality appraisal of systematic reviews (2016 version)**

| **Included study** | **Q1** | **Q2** | **Q3** | **Q4** | **Q5** | **Q6** | **Q7** | **Q8** | **Q9** | **Q10** | **Q11** |
| --- | --- | --- | --- | --- | --- | --- | --- | --- | --- | --- | --- |
| Yesilkaya et al. [2] | Yes | Yes | Yes | Yes | Unclear | Yes | Yes | Yes | No | Yes | Yes |
| Li, S. N. et al. [3] | Yes | Yes | Yes | Yes | Yes | Yes | Yes | Yes | Yes | Yes | Yes |
| Agarwal et al. [4] | Yes | Yes | Yes | Yes | Yes | Yes | Yes | Yes | No | Yes | Yes |
| Ağırkan et al. [5] | Yes | Yes | Yes | Yes | Unclear | Yes | Yes | Yes | Yes | Yes | Yes |
| MacKenzie et al. [6] | Yes | Yes | Yes | Yes | Unclear | Yes | Yes | Yes | Yes | Yes | Yes |
| Kulasinghe et al. [7] | Yes | Yes | Yes | Yes | Yes | Yes | Yes | Yes | Yes | Yes | Yes |
| Juvin et al. [8] | Yes | Yes | Yes | Yes | Unclear | Yes | Yes | Yes | No | Yes | Yes |
| Hartley et al. [9] | Yes | Yes | Yes | Yes | Yes | Yes | Yes | Yes | No | Yes | Yes |
| Merriman et al. [10] | Yes | Yes | Yes | Yes | Yes | Yes | Yes | Yes | Unclear | Yes | Yes |
| Yu et al. [11] | Yes | Yes | Yes | Yes | Yes | Yes | Yes | Yes | Yes | Yes | Yes |
| Rutherford et al. [12] | Yes | Yes | Yes | Yes | Yes | Yes | Yes | Yes | Unclear | Yes | Yes |
| Catalano et al. [13] | Yes | Yes | Yes | Yes | Yes | Yes | Yes | Yes | No | Yes | Yes |

**Notes:** Q1–Q11 correspond to the JBI critical appraisal criteria for systematic reviews, including clarity of the review question, appropriateness of inclusion criteria, adequacy of the search strategy, appraisal methods, data extraction, synthesis, assessment of publication bias, and support of conclusions by the presented data.

**Table S6. Evidence synthesis and grading**

| **Evidence category** | **Evidence content** | **Level of evidence** |
| --- | --- | --- |
| Rights | 1. Families of children with ASD and their family caregivers [FCs] should be provided with individualized information, resources, and psychological support tailored to family needs [1]. | 1a |
| Macro-level policy | 2. It is recommended that children with ASD and their FCs be incorporated into mental health support service systems provided by medical institutions to ensure effective protection of the well-being of both children and FCs [13]. | 1b |
| Establishment of multidisciplinary teams | 3. It is recommended that medical or special education institutions organize multidisciplinary teams involving physicians and psychologists to establish FC support groups [5, 10]. | 1b |
|  | 4. Healthcare professionals can serve as navigators for FCs, assisting them in accessing appropriate services and resources [10]. | 1b |
|  | 5. Psychologists can provide psychoeducation, social activities, and skills training to enhance FCs’ stress management, coping skills, and problem-solving abilities [5, 10]. | 1b |
| Intervention format | 6. Group-based interventions are recommended for FCs to help establish social support networks [5]. | 1b |
| Intervention setting | 7. It is recommended that FC support groups be established within child developmental rehabilitation and training institutions [10]. | 1b |
| Intervention type | 8. Interventions include two types: FC-mediated interventions and FC-focused interventions. FC-mediated interventions aim to manage children’s core symptoms and problem behaviors, whereas FC-focused interventions emphasize improving FCs’ health status, primarily including mindfulness-based interventions, acceptance and commitment therapy (ACT), cognitive behavioral therapy, psychoeducation programs, and expressive writing [3]. | 1a |
|  | 9. FC-mediated interventions may improve FCs’ parenting self-efficacy to some extent; however, they do not significantly improve caregiver burden or depressive symptoms [6]. | 1a |
|  | 10. FC-focused interventions are recommended, as they can significantly reduce FCs’ stress and depressive symptoms, markedly improve parent–child relationships, and have positive effects on children’s problem behaviors [3, 11]. | 1a |
|  | 11. It is recommended that FC-focused interventions be combined with parenting skills training [3]. | 1a |
|  | 12. The optimal duration of FC-focused interventions is 5–8 weeks [3]. | 1a |
| Mindfulness-based interventions | 13. Mindfulness-based interventions enhance FCs’ self-regulation of emotions and behaviors, significantly reducing stress levels [2~4, 10, 12], and can also markedly alleviate depressive symptoms [3, 13]. | 1a |
|  | 14. Mindfulness-based interventions help improve FCs’ overall health status [10]. | 1b |
|  | 15. Mindfulness-based interventions promote greater acceptance of children during daily caregiving and contribute to improved parent–child interactions [2]. | 1b |
|  | 16. Group-based therapy is an important adjunctive approach for cultivating mindfulness awareness among FCs [9]. | 1 |
|  | 17. Home practice should be flexibly adjusted according to FCs’ lifestyles, with efforts to reduce the time burden of practice and prioritize informal practice methods [9]. | 1 |
|  | 18. Integrating mindfulness practice into daily life is recommended to overcome difficulties associated with formal practice [9]. | 1 |
|  | 19. For FCs experiencing high stress and limited capacity for formal mindfulness practice, the use of smartphone-based mindfulness applications is recommended [9]. | 1 |
| Acceptance and commitment therapy [ACT] | 20. ACT enhances individuals’ self-awareness, self-acceptance, and self-regulation abilities, significantly reducing FCs’ stress and depressive symptoms [3, 13]. | 1a |
|  | 21. ACT also improves psychological flexibility, helping FCs accept caregiving realities that cannot be changed [8, 10]. | 1b |
|  | 22. Combining ACT with self-compassion interventions may further reduce stress levels [7]. | 1a |
| Psychoeducation programs | 23. Combined mindfulness-based interventions and ACT have significant positive effects on FCs’ mental health and children’s behaviors [3]. | 1a |
|  | 24. Group-based psychoeducation programs improve FCs’ parenting self-efficacy and reduce anxiety and depressive symptoms [5, 6]. | 1a |
|  | 25. Compared with parent-mediated interventions, psychoeducation programs have greater effects on improving FCs’ parenting self-efficacy [6]. | 1a |
|  | 26. Providing accurate and reliable information about ASD through psychoeducation facilitates the use of problem-solving skills [13] | 1b |
| Problem-solving skills training | 27. Problem-solving skills training helps FCs actively cope with daily caregiving challenges [10]. | 1b |
| Expressive writing | 28. Expressive writing interventions reduce FCs’ stress levels [3]. | 1a |
|  | 29. Expressive writing interventions also alleviate anxiety symptoms among FCs [13]. | 1b |
| Multicomponent interventions | 30. Combined gratitude-based interventions improve FCs’ psychosocial outcomes [11]. | 1b |
|  | 31. It is recommended to adopt multicomponent interventions incorporating two or more core components, such as psychoeducation, mindfulness-based interventions, ACT, and behavioral management, as they significantly improve FCs’ psychosocial outcomes [11]. | 1b |

**References**

1. National Institute for Health and Care Excellence. Autism spectrum disorder in under 19s: recognition, referral and diagnosis. London: NICE; 2011.

2. Yesilkaya M, Magallón-Neri E. Parental stress related to caring for a child with autism spectrum disorder and the benefit of mindfulness-based interventions for parental stress: A systematic review. SAGE Open. 2024;14(2): 21582440241235033. doi:10.1177/21582440241235033

3. Li SN, Chien WT, Lam SKK, Chen ZY, Ma X. Effectiveness of parent-focused interventions for improving the mental health of parents and their children with autism spectrum disorder: A systematic review and meta-analysis. Res Autism Spectr Disord. 2024;114: 102389. doi:10.1016/j.rasd.2024.102389

4. Agarwal R, Wuyke G, Sharma U, Burke SL, Howard M, Li T, et al. Stress and anxiety among parents of transition-aged children with autism spectrum disorder: A systematic review of interventions and scales. Rev J Autism Dev Disord. 2024;11: 320–342. doi:10.1007/s40489-022-00340-8

5. Ağırkan M, Koç M, Haskan Avcı Ö. How effective are group-based psychoeducation programs for parents of children with ASD in Turkey? A systematic review and meta-analysis. Res Dev Disabil. 2023;139: 104554. doi:10.1016/j.ridd.2023.104554

6. MacKenzie KT, Eack SM. Interventions to improve outcomes for parents of children with autism spectrum disorder: A meta-analysis. J Autism Dev Disord. 2022;52(7): 2859–2883. doi:10.1007/s10803-021-05164-9

7. Kulasinghe K, Whittingham K, Mitchell AE, Boyd RN. Psychological interventions targeting mental health and the mother–child relationship in autism: A systematic review and meta-analysis. Dev Med Child Neurol. 2023;65(3): 329–345. doi:10.1111/dmcn.15432

8. Juvin J, Sadeg S, Julien-Sweerts S, Zebdi R. Acceptance and commitment therapy for the parents of children and adolescents with autism spectrum disorder: A systematic review. J Autism Dev Disord. 2022;52(1): 124–141. doi:10.1007/s10803-021-04923-y

9. Hartley M, Due C, Dorstyn D. Barriers and facilitators to engaging individuals and families with autism spectrum disorder in mindfulness and acceptance-based therapies: A meta-synthesis. Disabil Rehabil. 2022;44(17): 4590–4601. doi:10.1080/09638288.2021.1921859

10. Merriman K, Burke T, O’Reilly G. A systematic review of the effectiveness and efficacy of clinician-led psychological interventions for parents of children with ASD. Res Autism Spectr Disord. 2020;76: 101584. doi:10.1016/j.rasd.2020.101584

11. Yu Y, McGrew JH, Boloor J. Effects of caregiver-focused programs on psychosocial outcomes in caregivers of individuals with ASD: A meta-analysis. J Autism Dev Disord. 2019;49(12): 4761–4779. doi:10.1007/s10803-019-04181-z

12. Rutherford M, Singh-Roy A, Rush R, McCartney D, O’Hare A, Forsyth K. Parent focused interventions for older children or adults with ASD and parent wellbeing outcomes: A systematic review with meta-analysis. Res Autism Spectr Disord. 2019;68: 101450. doi:10.1016/j.rasd.2019.101450

13. Catalano D, Holloway L, Mpofu E. Mental health interventions for parent carers of children with autistic spectrum disorder: Practice guidelines from a critical interpretive synthesis (CIS) systematic review. Int J Environ Res Public Health. 2018;15(2): 341. doi:10.3390/ijerph15020341
