# Supplementary material for: An evidence-informed psychosocial intervention program for caregiver burden among family caregivers of children with autism spectrum disorder: a multi-stage development and Delphi study
Source: PLoS One. 2026 Aug 12;21(8):e0354823. doi: 10.1371/journal.pone.0354823 (PMC13465810; doi:10.1371/journal.pone.0354823)
Supplement: Supplementary file 5 [file pone.0354823.s004.docx]

**S4 File. Finalized psychosocial intervention program for caregiver burden among family caregivers of children with ASD**

**Overview of the psychosocial intervention program**

This S4 File presents the finalized psychosocial intervention program developed through the multi-stage development process and two rounds of Delphi consultation. The program has not yet been implemented. Accordingly, the intervention procedures, outcome measures, and evaluation plans described below are intended for future feasibility and effectiveness testing.

a. Intervention purpose: This intervention program aims to alleviate caregiver burden through a comprehensive and integrated intervention approach, with the goal of improving the physical and psychological well-being and quality of life of family caregivers (FCs) of children with autism spectrum disorder (ASD).
b. Intervention personnel: The intervention team will consist of the researchers, psychiatrists or pediatricians, psychologists, nurses with qualifications in psychological counseling, and rehabilitation therapists. Among them, the researchers, psychologists, and nurses will be required to have received systematic training in mindfulness and/or Acceptance and Commitment Therapy (ACT) and to possess relevant instructional qualifications.
c. Target population: The intervention will target family caregivers (FCs) of children with ASD. Priority will be given to FCs whose children are only children, FCs who are female or unemployed, and FCs caring for children with moderate to severe ASD (severity level II or III).
d. Intervention format: The intervention will be delivered through a combination of face-to-face and online sessions. All sessions will be video-recorded to accommodate participants who are unable to attend due to unforeseen circumstances. The intervention will be delivered in groups of no more than 10 participants. For selected in-session activities, participants may be divided into small subgroups of 3–5 to facilitate discussion and practice.
e. Intervention schedule: The intervention will consist of eight sessions, delivered once per week over an eight-week period. Each session will last approximately 90–120 minutes. Where feasible, the intervention may be conducted concurrently with the child’s rehabilitation training.
f. Intervention setting: The intervention will be conducted in classrooms at special child rehabilitation centers or via online platforms.
g. Outcome measures: The primary outcome will be caregiver burden. Secondary outcomes will include psychological capital, perceived social support, illness cognition, affiliate stigma, coping styles, and ASD-related knowledge assessed using a structured questionnaire.
h. Pre-intervention assessment: Baseline data will be collected from FCs prior to the initiation of the intervention to assess their demographic characteristics and baseline status.
i. Post-intervention evaluation: Quantitative and qualitative data will be collected immediately after the intervention and at 1-, 3-, and 6-month follow-up time points.
j. Precautions: This intervention program is not intended to replace pharmacological or other medical treatments. Participants who are taking psychotropic medications should not discontinue medication without medical advice due to participation in this program. During sessions 2, 3, 5, and 6, if a participant exhibits excessive emotional distress, the intervention will be immediately suspended, and psychological support will be provided by a qualified psychologist.

**Pre-intervention preparation**

a. Participant recruitment and consent: Eligible participants will be recruited prior to intervention implementation. All participants will participate voluntarily and will provide written informed consent.
b. Relationship building

- Introduction of the intervention team: At the beginning of the program, the researchers will introduce the intervention team to participants, including team members’ names, roles, and responsibilities.
- Ice-breaking activity: Participants will be invited to introduce themselves using a structured outline, including a participant-selected pseudonym or study code, broad employment status, the child’s age group, approximate time since diagnosis, and general support expectations. This activity will aim to enhance mutual understanding and establish trust among participants.
- Establishment of an online communication group: An online WeChat group named “Starlight Caregivers Alliance” will be established, and participants will be invited to join. Spouses or other family members will also be encouraged to participate. According to the intervention schedule, practice-related audio recordings, videos, and electronic reading materials—including The Eight-Week Mindfulness Journey, ACT Made Simple: A Practical Guide to Acceptance and Commitment Therapy, and Self-Compassion: Embracing Imperfection—will be shared within the group to support practice. Homework assignments may be submitted either in the group or privately to the researchers in various formats, including text, images, videos, or voice messages.

c. Introduction of the intervention program: Before implementation, the purpose, content, format, duration, and specific tasks of the intervention program will be explained to participants.
d. Baseline assessment: Baseline data will be collected before the intervention using a demographic questionnaire, the Caregiver Burden Inventory, the Psychological Capital Questionnaire, the Perceived Social Support Scale, the Illness Cognition Questionnaire, the Affiliate Stigma Scale, the Simplified Coping Style Questionnaire, and an ASD-related knowledge questionnaire, in order to assess participants’ baseline characteristics.
e. Group allocation: Participants will be grouped on a voluntary basis. The overall intervention group size will not exceed 10 participants, and participants may be divided into small subgroups of 3–5 for selected discussion and practice activities. During intervention evaluation activities, each group will select a representative to speak on a rotating basis.

Finalized psychosocial intervention program: The detailed structure and content of the finalized psychosocial intervention program are presented in Table S7.

**Post-intervention summary**

The intervention has not yet been implemented, and all outcome measures and evaluation procedures described represent proposed plans for future feasibility and effectiveness studies.

a. Program summary: At the conclusion of the intervention, the researchers will summarize the intervention content and the overall learning process with participants.
b. Collection of quantitative data: Overall intervention effectiveness will be evaluated using the Caregiver Burden Inventory, the Psychological Capital Questionnaire, the Perceived Social Support Scale, the Illness Cognition Questionnaire, the Affiliate Stigma Scale, the Simplified Coping Style Questionnaire, and an ASD-related knowledge questionnaire.
c. Collection of qualitative data: Qualitative data will be collected through semi-structured interviews. Participants will be invited to share their experiences, perceived benefits, challenges encountered during the intervention, and suggestions for improving the intervention program.
d. Provision of follow-up support: Following completion of the intervention, the researchers will continue to provide professional information, resources, and support through the WeChat group.

**Table S7 Finalized psychosocial intervention program**

| **Module** | **Theme** | **Purpose and objectives** | **Intervention content** | **Format** | **Evaluation** | **Personnel** | **Schedule** |
| --- | --- | --- | --- | --- | --- | --- | --- |
| 1. Stressors | 1.1 Sources of stress among FCs | Purpose: To help FCs accurately identify the sources of stress they face and to develop a preliminary understanding of ASD-related stressors. Objective: FCs are able to identify specific stressors they experience, understand the psychological processes through which stressors exert effects, and recognize common stress-related concerns. | 1.1.1 Psychological processes through which stressors affect individuals *Knowledge-based instruction:* Introduction to specific stressors faced by FCs, the psychological processes through which stressors exert their effects, and differences in cognitive and coping responses to stressors.  1.1.2 Basic knowledge of ASD *Knowledge-based instruction:* Etiology and pathogenesis of ASD, clinical manifestations, diagnosis, treatment, and key points of care. | Lecture (online live session with recorded playback) | Knowledge assessment to evaluate whether FCs can correctly identify stressors and master basic ASD-related knowledge | Researchers, pediatricians, psychiatrists | Week 1 90–120 min |
| 2. Cognitive appraisal | 2.1 Expectations regarding illness prognosis | Purpose: To help FCs develop realistic expectations regarding their child’s illness prognosis. Objective: FCs are able to form expectations that are aligned with the actual prognosis of ASD. | 2.1.1 Introduction to knowledge of illness prognosis *Knowledge-based instruction:* Basic information on prognosis of ASD.  2.1.2 Clarifying FCs’ expectations regarding illness prognosis *In-class activities:* Small-group discussion: FCs share their expectations regarding their child’s prognosis. Classroom sharing: Group representatives summarize and present discussion results. Facilitator-guided discussion strategy: “Please first list the minimum realistic expected outcome (to reduce expectations and enhance a sense of achievement), then gradually raise expectations by clarifying evidence-based possibilities and feasibility, until reaching the best achievable prognosis for the child.” | Lecture; group discussion; experience sharing | Group representatives present discussion outcomes to assess whether FCs are able to form realistic expectations regarding prognosis |  |  |
| 2. Cognitive appraisal | 2.2 Psychological capital | Purpose: To enhance psychological capital among FCs. Objectives: (1) FCs are able to correctly understand basic concepts of mindfulness and accept  mindfulness-based approaches. (2) FCs are able to correctly apply mindfulness techniques, including mindful breathing, body scan, and mindful eating. | 2.2.1 Introduction to mindfulness-based intervention [1]  (1) Knowledge-based instruction: 1) Mindfulness: Mindfulness is defined as purposeful, present-moment, and non-judgmental awareness. It emphasizes direct awareness of current experiences rather than becoming absorbed in thoughts about the past or future. 2) Three core characteristics: a. Present-moment attention: The core of mindfulness lies in focusing on the present moment, thereby reducing rumination about the past and anxiety related to uncertainty about the future. Through practicing present-moment attention, individuals can  perceive moment-to-moment details more clearly and reduce the intrusion of negative emotions. b. Acceptance: Mindfulness emphasizes acceptance of all emotional experiences, whether positive, negative, or neutral. FCs are encouraged to allow emotions to exist rather than suppressing or avoiding them. Through acceptance, individuals learn to coexist with emotions and reduce internal conflict and distress. c. Awareness: Mindfulness practice enhances self-awareness, enabling individuals to become more sensitive to changes in emotions, thoughts, and bodily sensations. By strengthening awareness, FCs can better recognize their emotional and psychological states and respond to stressors with greater calmness and balance. 3) Types of mindfulness practices: Mindful breathing, body scan, mindful eating, mindful stretching, and emotional awareness. 4) Effects: a. Improvement of emotional regulation: Mindfulness helps individuals enhance emotional awareness and recognize emotions in a timely manner, thereby selecting appropriate strategies for emotional regulation. Through acceptance of emotional experiences, individuals learn to coexist with emotions in a more balanced and peaceful manner.  b. Reduction of stress and anxiety Mindfulness practice may effectively reduce the negative impact of daily stress and anxiety. By focusing on the present moment and accepting emotional experiences, FCs can avoid excessive rumination about future uncertainties or past failures and gradually cultivate flexibility and resilience in coping with stress. Mindfulness practice may also promote physical relaxation, reduce physiological stress responses, and support overall physical and psychological well-being. (2) Guided discussion Facilitator-guided reflection: “Have you ever felt more exhausted due to anxiety or self-blame? Would you be willing to try a new approach—observing and accepting your internal states with kindness rather than becoming trapped in negative emotions?” (3) Case sharing: mindfulness in daily life Case 1: Hypothetical Caregiver A is the mother of a 3-year-old child with ASD. She spends most of her day accompanying her child during rehabilitation training and continues to organize various training plans at night. She often feels physically and emotionally exhausted and occasionally experiences emotional breakdowns. After learning mindful breathing, she pauses when feeling anxious and practices deep breathing for three minutes, focusing her attention on the breath rather than being driven by distressing thoughts. She reports that she is able to calm down more quickly and that the frequency of emotional breakdowns has decreased. Case 2: Hypothetical Caregiver B is a full-time father who accompanies his child to classes and conducts rehabilitation training at home on a daily basis. While waiting for his child, he often uses his mobile phone, which paradoxically increases his anxiety. He begins to practice mindful walking during waiting periods—walking slowly, feeling the contact of each step with the ground, and observing the surrounding environment rather than immersing himself in his phone or anxious thoughts. He reports feeling calmer and becoming more attentive to changes in his child’s emotions, which has helped improve the parent–child relationship. | Lecture; discussion | Assessment of whether FCs are able to correctly understand and apply mindfulness-based techniques, thereby | Researchers, psychologists, nurses | Week 2 90–120 min |
|  |  |  |  |  | enhancing psychological capital  If FCs fail to complete daily check-ins or provide feedback, or if submitted content is inaccurate, researchers may provide reminders via the WeChat group |  |  |
| 2. Cognitive appraisal | 2.2 Psychological capital |  | 2.2.2 Mindful breathing (1) Key points for practice FCs are reminded not to deliberately change the speed or depth of breathing, but to observe the breath naturally. If attention becomes distracted or emotions fluctuate, FCs are encouraged to gently acknowledge these experiences rather than forcing themselves to “calm down.” (2) Guided in-session practice FCs are instructed to choose a comfortable position, sitting or lying down, and to close their eyes. They are guided to focus attention on their breathing, noticing each inhalation and exhalation, sensing the flow of air entering the nasal cavity and chest and then slowly leaving the body. When attention naturally wanders, FCs are gently guided to bring attention back to the breath without self-judgment or self-criticism. | Lecture; demonstration; practice |  | Researchers, psychologists, nurses | Week 2 90–120 min |
|  |  |  | 2.2.3 Body scan (1) Key points for practice If discomfort is felt in any part of the body, FCs are encouraged not to eliminate the sensation immediately, but to gently observe and accept the current bodily state. Complete relaxation during each body scan is not required, as relaxation is a gradual process. Over time, the body may gradually adapt and release tension. (2) Guided in-session practice FCs are guided to find a comfortable position, sitting or lying down, and to close their eyes. Starting from the feet, attention is gradually directed to different parts of the body, including the feet, calves, thighs, abdomen, chest, back, shoulders, arms, hands, fingers, neck, and head. FCs are guided to notice sensations of tension, relaxation, or discomfort in each area without judgment, simply allowing sensations to be present. When attending to each body part, FCs may silently express gratitude toward the body and gently say to themselves, “Thank you for your effort.” | Lecture; demonstration; practice |  |  |  |
|  |  |  | 2.2.4 Mindful eating (1) Key points for practice FCs are encouraged to avoid multitasking and to focus on the food and the process of eating, minimizing external distractions as much as possible. When attention becomes distracted, FCs are gently guided to bring their attention back to the present-moment sensations of eating. (2) Guided in-session practice FCs are instructed to take a raisin or another small piece of food and carefully observe its appearance, color, and texture. Using their fingers, they are guided to notice the surface and contours of the food. FCs are then guided to bring the food close to the nose and gently notice its aroma. Next, they slowly place the food into the mouth, noticing its texture and taste, and paying attention to subtle changes during chewing. The eating pace is intentionally slowed, with attention focused on the sensations associated with each bite and swallow. Through mindful awareness of each detail of the eating process, FCs cultivate sustained attention toward food and eating.  Homework for Week 2 FCs are instructed to follow the video materials shared in the WeChat group, including practices of mindful breathing, body scan, and mindful eating. At least one of the listed practices should be completed once per day. Upon completion, FCs are encouraged to check in within the WeChat group and share their practice experiences. Check-in template: (1) Time periods available for practice during the week; (2) personal experiences, observations, and questions (if any). | Lecture; demonstration; practice | Review |  |  |
| 2. Cognitive appraisal | 2.2 Psychological capital | Purpose: To enhance FCs’ psychological capital. Objectives: (3) FCs are able to appropriately apply mindfulness-based practices, including mindful stretching, emotional awareness, and mindful walking. (4) FCs are able to articulate key points requiring attention during practice. | Warm-up and review Practice of body scan; 1–2 FCs are invited to share their daily mindfulness experiences; group discussion focuses on characteristics of attention; the facilitator analyzes common difficulties encountered during mindfulness practice and provides targeted suggestions. | Sharing; discussion | Via WeChat group check-ins and shared practice frequency and content  If FCs fail to check in on time or provide inaccurate feedback, the researcher may send reminders via WeChat | Researchers; psychologists; nurses | Week 3 90–120 min |
|  |  |  | 2.2.5 Mindful stretching (1) Key points for practice During stretching, FCs are encouraged to maintain natural and smooth breathing and to avoid holding the breath or exerting excessive force. If discomfort arises, FCs may appropriately reduce the intensity of the stretch or relax the body, without forcing themselves. (2) Guided in-session practice FCs are instructed to stand and gradually perform stretching movements involving different body parts (e.g., arms, back, waist, and legs). During each movement, attention is directed toward sensations of muscle stretch and bodily relaxation. FCs are encouraged to remain attentive to the sensations associated with each movement, avoid overexertion, and synchronize stretching with mindful breathing. Through mindful awareness of bodily stretching, FCs are guided to relax muscles and alleviate physical tension. | Lecture; demonstration; practice |  |  |  |
|  |  |  | 2.2.6 Emotional awareness (1) Knowledge-based explanation: sources of emotional distress and coping strategies Sources: Children’s symptoms and problem behaviors; the intensity and time demands of daily caregiving; insufficient social support; financial strain; worries about the child’s future; and discrepancies between self-expectations and reality. Coping strategies: a. Awareness and acceptance of emotions. During the caregiving process, FCs may experience a range of emotions, such as anxiety, sadness, and anger. Mindfulness practice helps FCs become aware of the emergence of these emotions and understand that emotional fluctuations represent normal responses to caregiving demands and life challenges, rather than signs of failure or personal inadequacy. | Lecture; demonstration; practice |  |  |  |
|  |  |  | Allowing and accepting emotions FCs are encouraged to allow themselves to experience these emotions, rather than suppressing or ignoring them. Mindfulness practice helps FCs approach emotions with a nonjudgmental attitude and recognize that emotions themselves do not need to be controlled; instead, it is the excessive reactions or maladaptive behaviors driven by emotions that require regulation. When emotions arise, mindfulness creates a buffer space for FCs, allowing them to pause briefly, observe their emotions, and refrain from responding impulsively. In this way, FCs are able to respond more calmly, rationally, and constructively. b. Focusing on the present moment Mindfulness helps FCs bring their attention to the present moment and avoid excessive immersion in past regrets or worries about the future. It facilitates awareness of emotional reactions that arise during caregiving and enhances FCs’ capacity for emotional observation and regulation. When emotions emerge, FCs can use breathing or other mindfulness practices to adjust their internal state and face challenges with a more balanced mindset. (2) Key points for practice During practice, emotions may intensify. At such times, FCs are encouraged not to attempt to suppress emotions, but to allow themselves to fully experience the emotional process. There is no need to analyze emotions; FCs are guided to simply observe their presence and attend to their fluctuations and changes. (3) Guided in-session practice FCs are guided to recall their emotional state at the time of the child’s initial diagnosis and to focus on those feelings. They are instructed to observe bodily changes (e.g., increased heart rate, rapid breathing) and emotional fluctuations (e.g., anger, anxiety). Without judging emotions as good or bad, FCs are guided to bring attention to the breath, accept the presence of emotions, and allow themselves to remain with these emotions without attempting to change them. This practice helps FCs become aware of their current emotional state and reduces the likelihood of being controlled by emotions. |  |  |  |  |
|  |  |  | 2.2.7 Mindful walking (1) Key points for practice FCs are encouraged to avoid exaggerated or hurried movements, slow down their pace, and maintain steady breathing. When attention becomes distracted, FCs are gently guided to bring attention back to the steps, without forcing themselves to “concentrate.” (2) Guided in-session practice FCs are instructed to stand in a suitable space and experience walking with a sense of “groundedness.” They are guided to notice the sensations of the feet contacting and lifting from the ground, observe how body weight shifts during movement, and become aware of the surrounding environment, such as airflow and changes in sound. These sensations are observed without judgment. By attending to each step during walking, FCs are guided to remain present in the moment and reduce mental distraction.  Homework for Week 3 FCs are instructed to follow the video materials shared in the WeChat group, including practices of mindful stretching, emotional awareness, and mindful walking. At least one of the listed practices should be completed once per day. Upon completion, FCs are encouraged to check in within the WeChat group and share their practice experiences. Check-in template: (1) Time periods available for practice during the week; (2) personal experiences, observations, and questions (if any). | Lecture; demonstration; practice | Review |  |  |
| 2. Cognitive appraisal | 2.3 Perceived social support | Purpose: To enhance FCs’ perceived social support. Objectives: (1) Through sharing gratitude experiences, FCs’ perceived level of social support can be enhanced. (2) FCs are able to express gratitude toward relatives and friends through expressive writing. | Warm-up and review Practice of mindful stretching; 1–2 FCs are invited to share their daily mindfulness experiences; group discussion focuses on characteristics of attention; the facilitator analyzes difficulties encountered during mindfulness practice and provides targeted suggestions.  2.3.1 Gratitude-based intervention [2] (1) Knowledge-based explanation Gratitude: Gratitude refers to recognizing the good things that others do for oneself and appreciating the intention behind such help. Gratitude-based intervention aims to intentionally cultivate a grateful mindset, helping FCs more clearly recognize support and assistance in daily life, strengthen trust in and reliance on others, reduce negative emotions, and enhance awareness of support from family members, friends, and society, thereby improving FCs’ perceived social support. (2) In-session activities Small-group discussion: FCs share their personal gratitude experiences. Class discussion: Group representatives share their gratitude experiences. The facilitator guides discussion on how gratitude helps FCs enhance their perception of social support. Guiding questions: “During daily caregiving, have you ever felt grateful for help from family members, friends, or healthcare professionals? How did this feeling of gratitude affect your emotions?” “How can expressing gratitude strengthen your perception of social support? Are there any concrete examples?” Points for attention: Some FCs may feel that “I do not receive enough support” or “I do not deserve such support.” In such cases, FCs are guided to gradually recognize small forms of support in everyday life. | Sharing; discussion; lecture | Whether FCs are able to demonstrate specific supportive behaviors and emotional responses related to social support in expressive writing | Researchers | Week 4 90–120 min |
|  |  |  | 2.3.2 Expressive writing Writing practice FCs are instructed to write a letter to their child, partner, friend, or healthcare professional, focusing on one or more specific actions that made them feel warmth and gratitude. Attention is directed toward concrete supportive behaviors rather than abstract feelings of gratitude alone. Examples of concrete behaviors: “Thank you for accompanying us to the hospital when my child was ill.” Examples of emotional responses: “Because of your companionship, I felt less afraid.” The emphasis is not on writing technique, but on the authentic expression of emotions and appreciation of supportive behaviors. Points for attention FCs are encouraged to write freely without restrictions on word count or format and to describe their emotions and experiences in as much detail as possible. During writing, FCs are advised not to engage in self-evaluation or analysis, but to focus on the natural flow of emotions.  Homework for Week 4 “Three good things” exercise: FCs are instructed to record three events during the week for which they feel grateful and to share these records during the following session. Recording content: (1) Specific events: Each day, FCs reflect on positive or gratitude-evoking experiences, which may include help from others, small personal achievements, or positive moments in daily life. (2) Emotional responses: FCs reflect on the meaning of these positive events and their emotional reactions (e.g., “I felt especially warm when my friend stayed with me for an hour”). (3) Emotional impact: FCs reflect on how these events influenced their emotions and helped them respond more positively to everyday challenges. | Writing; recording; reflection |  |  |  |
| 2. Cognitive appraisal | 2.4 Illness cognition | Purpose: To enhance FCs’ level of illness cognition (reduce feelings of helplessness, accept reality, and cultivate a sense of meaning). Objectives: (1) FCs are able to accept the perspective that “suffering is inevitable, and it is possible to live a meaningful life despite suffering.” (2) FCs are able to accept negative cognitions and emotions without being dominated by them. | Warm-up and review Practice of emotional awareness; 1–2 FCs are invited to share their gratitude records and experiences from the previous week; the facilitator guides discussion on the impact of gratitude and writing on psychological functioning.  2.4.1 Introduction to Acceptance and Commitment Therapy (ACT) [3] (1) Knowledge-based explanation 1) Problem elicitation: “During the caregiving process, have you noticed that attempts to avoid anxiety and helplessness often lead to greater efforts at control, which paradoxically intensify emotional distress?” Traditional coping strategies often involve avoidance, suppression, or forced control of negative emotions; however, such approaches may result in emotional rebound and even exacerbate distress. 2) ACT: While mindfulness helps FCs attend to the present moment and observe emotions with acceptance, ACT focuses on changing the relationship between FCs and their negative cognitions and emotions. Through ACT, negative thoughts and emotions no longer dominate behavior; instead, FCs learn to coexist with them. 3) Core principles of ACT: Suffering is inevitable, and emotions such as anxiety, helplessness, and regret are normal responses. Struggling against suffering often intensifies it. The key lies in learning how to live with suffering while still pursuing a meaningful life. 4) The six core processes of ACT: a. Acceptance: Accepting emotions rather than resisting them. b. Cognitive defusion: Recognizing thoughts as thoughts, not facts. c. Present-moment awareness: Cultivating mindfulness and reducing excessive focus on the past or future. d. Self-as-context (self-observation): Recognizing that the self is larger than one’s emotions and thoughts. e. Values clarification: Identifying what truly matters. f. Committed action: Acting in accordance with values rather than being constrained by emotions.  Key emphasis “ACT does not ask us to endure suffering; rather, it helps us learn how to continue living, growing, and finding meaning in the presence of suffering.” (2) In-session activities Paper ball exercise: Each FC is given a sheet of paper and asked to crumple it into a ball and hold it tightly in the palm. FCs are instructed to grip the paper ball as firmly as possible for 30 seconds, noticing tension and discomfort in the hand. They are then asked: “If you keep gripping it all day, could you do it?” (Most participants respond that they could not.) FCs are then instructed to open their hand and release the paper ball, observing changes in bodily sensations. Facilitator-guided discussion “When you try hard to control anxiety and helplessness—like gripping the paper ball—do you become even more exhausted? When you let go, you may find that you can still continue doing other things. Trying to control emotions is like gripping the paper ball and makes us more tired. If we learn to let go—rather than suppress emotions and allow them to exist naturally—we often feel much more relaxed. This is what acceptance means in ACT. Acceptance does not mean giving up; it means not being constrained by suffering.” | Sharing; discussion; lecture | Based on completion of weekly assignments and whether FCs demonstrate gradual acceptance of negative cognitions and emotions, and recognition of a “larger self” | Researchers; psychologists; nurses | Week 5 90–120 min |
|  |  |  | 2.4.2 Acceptance and cognitive defusion training (1) Acceptance training 1) Key points for practice When FCs are accustomed to suppressing emotions, facilitators remind them that acceptance does not mean liking emotions. Rather, emotions are treated as temporary experiences that do not define one’s entire life. FCs are encouraged to gradually step back from emotions, rather than attempting complete detachment from the outset. Each practice session is intended to gently enhance emotional regulation capacity.  (2) Guided in-session practice FCs are guided to sit comfortably, close their eyes, take deep breaths, and relax their bodies. They are then invited to recall a recent situation that evoked anxiety, regret, or feelings of helplessness. FCs are instructed not to attempt to change or suppress these emotions, but simply to observe their presence. FCs are guided to notice how these emotions manifest in the body (e.g., increased heart rate, rapid breathing, tightness in the shoulders). They are encouraged to allow themselves to fully experience these emotions and to silently tell themselves: “I allow these emotions to be present and fully accept them, rather than resisting them.” (3) Knowledge-based explanation 1) Emotional acceptance and cognitive defusion: Emotional acceptance refers to accepting emotions themselves, whereas cognitive defusion involves recognizing that the thoughts and beliefs underlying emotions are not equivalent to reality. Emotional acceptance helps FCs stop resisting emotions, while cognitive defusion enables them to recognize that thoughts associated with emotions are not facts, thereby allowing greater flexibility in responding. Through acceptance, FCs no longer avoid or suppress emotions; through cognitive defusion, they are less constrained by negative thoughts and can respond to stress more flexibly. 2) Cognitive defusion: FCs are guided to learn to maintain a certain distance from negative cognitions and to recognize that these cognitions are merely thoughts in the mind rather than accurate reflections of reality. For example, when the thought “I will never be able to do this” arises, FCs are guided to recognize: “This is just a thought, not a fact.” The core of defusion lies in not believing every thought that emerges. Through this process, FCs can choose not to be controlled by negative cognitions, reduce emotional distress driven by such thoughts, avoid vicious cycles between emotion and behavior, and thereby enhance emotional regulation capacity.  FCs may hold the belief “I am not capable of taking good care of my child,” which can lead to feelings of helplessness and influence behavior. However, if FCs learn to view this belief as a cloud passing by, they can recognize that it is only a temporary thought rather than a permanent state. (3) Cognitive defusion training Guided in-session practice a. Leaves on a stream exercise: FCs are guided to close their eyes, take deep breaths, and relax their bodies. They are invited to imagine themselves sitting beside a stream, watching leaves floating on the water. Each time a negative thought arises (e.g., “I am not good enough” or “I cannot take care of my child”), FCs are instructed to place that thought on a leaf and observe it gently floating downstream. These negative thoughts, like leaves, may appear quickly but will gradually drift away with the current. FCs are not required to grasp or hold onto them, but to allow them to pass naturally. b. Radio metaphor: FCs are guided to keep their eyes closed, remain relaxed, and breathe deeply. They are invited to imagine that the negative cognitions in their mind are like a radio broadcast playing in the background, with messages such as “I cannot do this” or “My child will never recover.” FCs are guided to imagine turning down the volume of this broadcast rather than following or arguing with it. They are encouraged to recognize that the broadcast is merely a signal in the mind and not an objective fact. When the sound becomes intrusive, FCs can gently remind themselves: “This is just a thought. I do not have to accept it, and I do not need to follow it.” | Lecture; demonstration; practice |  |  |  |
|  |  |  | 2.4.3 Self-observation (1) Knowledge-based explanation Self-observation: FCs are guided to view themselves from a third-person perspective, rather than defining themselves solely through emotions, behaviors, or self-evaluations. For example, FCs may perceive themselves as “failed parents”; however, in reality, they are experiencing emotions such as anxiety or helplessness. These emotions and thoughts do not define who they are as a person. When FCs recognize that they are experiencing emotions, thoughts, and life challenges—rather than being defined by them—negative emotions exert less control over behavior, enabling more rational and effective choices. Self-observation helps FCs understand: “I am not my emotions or thoughts; I am an individual capable of transcending them.” (2) Guided in-session practice Mountain-top perspective exercise: FCs are guided to sit upright, close their eyes, take deep breaths, and relax their bodies. They are invited to imagine themselves standing on the top of a mountain, overlooking a broad landscape that includes their past experiences, current circumstances, and possible futures. FCs are guided to reflect on challenges they have faced, emotional fluctuations, and moments of hardship, while also noticing how they have repeatedly overcome difficulties and continued moving forward. From this elevated perspective, FCs are no longer merely “caregivers driven by emotions,” but individuals who have persisted and remained resilient through various challenges. These experiences constitute their strength rather than being defined by momentary emotions, thoughts, or self-judgments. When facing emotional distress or cognitive challenges, FCs are encouraged to revisit this mountain-top perspective to recognize a broader and more resilient sense of self. | Lecture; demonstration; practice |  |  |  |
|  |  |  | Fifth-week assignment FCs are instructed to review the video materials shared in the WeChat group during the week, including practices related to emotional acceptance, cognitive defusion, and self-observation. FCs are encouraged to complete at least one practice per day. After each practice, they are asked to check in within the WeChat group and share their practice experiences. Check-in template: a. Time period available for daily practice during the week; b. Personal feelings, insights, and difficulties encountered (if any). FCs are further guided to reflect on and respond to three questions within the WeChat group: (1) Do you agree with the idea that “attempting to control emotions may paradoxically intensify emotional reactions”? (2) During practice, are you able to view negative cognitions as temporary “thoughts” rather than facts? (3) Are you able to reduce the influence of negative emotions on self-identity and perceive yourself as an independent individual capable of coping with challenges? Please explain your reasons. | Reflection; recording |  |  |  |
| 2. Cognitive appraisal | 2.4 Illness cognition | Purpose: To enhance FCs’ level of illness cognition (reducing helplessness, fostering acceptance of reality, and promoting a sense of meaning). Objective: (4) FCs are able to translate their core personal values into concrete actions, thereby enhancing their sense of control and meaning in life. | Warm-up and review: Practice emotional acceptance and cognitive defusion; invite 1–2 FCs to share their experiences of applying ACT practices in daily life and difficulties encountered. The facilitator guides group discussion and provides feedback and problem-solving suggestions.  2.4.4 Values clarification (1) Knowledge-based instruction: Values refer to what individuals consider most important in life and represent the deeper meaning and direction of living. Values are not external goals or outcomes, but guiding principles for how one lives and acts. For example: “As an FC, you may believe that your greatest value lies in providing care and companionship for your child, or in becoming a patient and supportive parent who facilitates your child’s growth, rather than merely completing caregiving tasks.” During the caregiving process, FCs often face intense stress and emotional distress. By clarifying personal values, FCs can draw inner motivation to persist through difficulties. Key message: Even in the face of hardship, values can serve as an internal anchor that supports FCs to continue moving forward. (2) Guided in-class practice: Values clarification worksheet: FCs are asked to reflect on their caregiving experiences and respond to the following guiding questions: a. During caregiving, what matters most to you? b. When do you feel most satisfied or proud, and what actions led to these feelings? What values are reflected in these actions? c. As a parent, what qualities or values do you hope your child will learn from you? d. What kind of caregiver do you hope to become? e. How do you hope your caregiving approach will have a positive impact on your child? FCs are given time to write down their responses to help clarify the values that best represent their core beliefs.  (2) Values Mapping  FCs are instructed to draw a *values map* on paper, writing “caring for my child” or “parental role” at the center. They are asked to list the values they consider most important during the caregiving process (e.g., love, patience, support, respect) and place these values in different directions on the map. The facilitator guides FCs to reflect on how these values can be enacted in daily life. For example, how can patience be expressed through concrete behaviors so that the child feels supported? FCs are encouraged to connect each value with specific caregiving actions and to reflect on the behavioral expressions underlying each value. For instance: *“If patience is one of my core values, I may respond to my child’s behavioral challenges with greater understanding rather than rushing to solve the problem.”*  (3) Key Practice Points  Some FCs may find it difficult to clearly identify their core values at first. They are encouraged to reflect on past moments when they felt proud, fulfilled, or deeply engaged in life, or on experiences that prompted deeper reflection. Through these recollections, FCs may gradually clarify their personal values. FCs may also perceive that acting in accordance with values is difficult. It is emphasized that value-based action is not achieved overnight; rather, each small and consistent step represents progress toward valued living.  (4) Guided Group Discussion  Discussion prompts**:** a. During the process of clarifying your values, did you identify deeper sources of motivation that you had not previously recognized? b. Can you view caring for your child as a form of value-driven action, rather than merely the completion of tasks? c. Based on your values map, can you clearly identify how you are living out these values in your daily life? | Sharing; discussion; lecture; practice | Classroom discussion and completion of weekly assignments; observation of whether FCs are able to translate core values into concrete actions | Researcher; psychologist; nurse | Week 6 90–120 min |
|  |  |  | 2.4.5 Values and Committed Action(1) Key concept explanation:Committed action refers to engaging in concrete behaviors that are consistent with one’s identified values after clarifying those values. These actions do not need to be large changes but rather small, value-consistent steps in daily life. FCs may abandon what truly matters due to anxiety or exhaustion. Committed action supports FCs in continuing meaningful behaviors despite the presence of negative emotions, thereby enhancing positive life experiences.(2) Committed action practice:a. Values–action matching: FCs review core values identified during values clarification (e.g., patience, love, support) and reflect on how these values can be enacted through daily behaviors. FCs complete a worksheet and select one to two specific actions as committed actions for the coming week.b. Micro-commitment exercise: FCs choose a small, feasible action and make a personal commitment using the format: “In the coming week, I commit to doing ___ (specific action) at least once per day in order to live out my value of ___ (core value).” Examples are provided (e.g., hugging the child daily to express love; pausing for one minute before responding to emotional outbursts to practice patience). | Lecture  Demonstration  Practice  Discussion | Review of weekly assignments  Observation of value-consistent behaviors |  |  |
|  |  |  | (3) Key practice guidance: FCs are encouraged to start with small, feasible actions and to recognize that failure does not indicate the end of the process. Missing a day or not completing a committed action does not mean that continuation is impossible. Living in accordance with one’s values is a long-term, cumulative process rather than a short-term change.(3) Guided discussion: Guiding questions include: What difficulties might arise when practicing committed actions? When persistence becomes challenging, how can FCs adjust their approach rather than give up entirely? Are there ways to remind oneself to engage in committed actions (e.g., setting reminders, writing notes, placing visual cues)?Key message: Failure does not represent termination but an opportunity for adjustment. If an action cannot be completed, its difficulty can be reduced rather than abandoning it altogether. Even small actions, when sustained, are more meaningful than occasional large efforts.  Week 6 homework: FCs follow the video content shared in the WeChat group, including emotional acceptance, cognitive defusion, and self-as-context exercises. Each day, at least one exercise is completed once, followed by check-in and sharing of experiences in the group. Check-in template: Time period available for practice each day. Personal feelings, insights, or difficulties encountered (if any).FCs are asked to reflect in the WeChat group on the following questions:• Have you developed strategies to overcome barriers to committed action?• What are these strategies, and why did you choose them? | Lecture Practice Discussion |  |  |  |
| 2. Cognitive Appraisal | 2.5 Affiliate Stigma | Objective: To reduce the level of affiliate stigma among FCs Goals:(1) FCs are able to accurately describe the three core components of self-compassion and correctly distinguish self-compassion from self-pity;(2) FCs are able to appropriately apply self-compassion meditation incorporating guided imagery and self-compassionate dialogue techniques to reduce self-criticism and stigma related to caregiving for children with ASD  — | Warm-up and review: Practice emotional acceptance and cognitive defusion; invite 1–2 FCs to share their experiences and challenges encountered during committed action practice. The facilitator leads a group discussion and provides problem-solving suggestions  2.5.1 Introduction to Self-Compassion [4] (1) Knowledge-based teaching:1) Self-compassion: A method of reducing self-criticism and feelings of shame by responding to oneself with understanding, kindness, and care.2) Three core components of self-compassion :a. Self-kindness: Responding to oneself with warmth and understanding rather than harsh self-judgment when facing difficulties. b. Common humanity: Recognizing that suffering and challenges are universal experiences, and that FCs are not alone. c. Mindfulness: Observing one’s emotions and thoughts in a balanced and objective manner rather than becoming immersed in them.3) Self-compassion vs. self-pity: Self-compassion involves accepting one’s pain with understanding, kindness, and a non-judgmental attitude, thereby promoting emotional regulation and personal growth. In contrast, self-pity reflects excessive focus on one’s suffering, immersion in negative emotions, lack of proactive action, and may hinder personal development.(2) Classroom activities: Small-group discussion: FCs share their personal experiences of affiliate stigma encountered in daily life. Group discussion: representatives summarize group discussions and share experiences with the whole group. The facilitator guides discussion using prompts such as: “Have you ever felt ashamed or blamed due to external evaluations while caring for your child?” “How have these feelings influenced your caregiving behaviors?” | Sharing  Discussion  Lecture | Evaluation through group representatives’ sharing of experiences related to self-compassion dialogue practice, reflecting FCs’ ability to correctly apply self-compassion strategies to reduce affiliate stigma | Researcher | Week 7  90–120 min |
|  |  |  | 2.5.2 Self-compassion meditation incorporating guided imagery (1) Key points for practice: FCs are reminded to maintain awareness and acceptance during imagery practice, without judging or excessively analyzing their emotions. They are encouraged to gently observe and allow emotional experiences to flow naturally, fostering a sense of safety and comfort.(2) Guided in-class practice:1) Self-compassion meditation incorporating guided imagery: FCs are invited to sit comfortably, close their eyes, relax their bodies, take deep breaths, and progressively release muscle tension. They are guided to recall a recent moment in which they felt shame or self-blame, particularly in situations related to caring for their child. FCs are encouraged to gently return to that moment, notice the associated emotions of shame or self-blame, and attend to bodily responses such as tension, heaviness, or discomfort. They are then guided to softly say to themselves: “I understand how you feel. These emotions are completely understandable, and many FCs experience similar struggles. You do not need to blame yourself. Allow yourself to feel these emotions and offer yourself more understanding and kindness. Regardless of the challenges you face, your efforts and dedication deserve recognition. You are worthy of love.”2) Imagery for releasing affiliate stigma: FCs are guided to sit comfortably, close their eyes, breathe deeply, and relax their bodies. They are asked to imagine their sense of affiliate stigma as a cloud of gray mist gently floating in their mind. With a warm and caring attitude, FCs imagine using their warm hands to softly hold this mist, allowing it to gradually become transparent and slowly dissipate. They are guided to say to themselves: “I acknowledge my feelings. Stigma is not my essence. I am worthy of love, understanding, and acceptance.” FCs are encouraged to experience the warmth and release associated with this imagery, allowing themselves to let go of burdens in this moment and to accept themselves as devoted FCs. Perfection does not exist; only love and acceptance remain. | Lecture  Demonstration  Practice |  |  |  |
|  |  |  | 2.5.3 Self-Compassion Dialogue (1) Key points for practice: Participants work in pairs, taking turns to switch roles, allowing each individual to experience both being a caregiver and being cared for. FCs are encouraged to extend the compassion and support experienced during the dialogue into daily life and to adopt a similar compassionate attitude toward themselves.(2) In-class activities: Simulated dialogue scenario: One participant plays themselves, while the other plays their inner supporter. Using self-compassionate language, they engage in a dialogue characterized by warmth and support. For example: “Although I feel helpless right now, I understand that this is a very difficult moment. I have tried my best, and this situation does not define me. I deserve more care and understanding.” Each group selects one participant to share their practice experience, with reflections guided by the three core components of self-compassion. Seventh-week assignment: FCs are asked to write a letter to themselves, focusing on their experiences of affiliate stigma and expressing understanding and compassion toward themselves. The tone of the letter should resemble that used when writing to a close friend, maintaining warmth and kindness while offering self-support. Self-compassion writing is framed as a gradual process of cultivating self-kindness rather than an immediate emotional shift. | Lecture  Practice  Sharing | Writing |  |  |
| 3. Coping | 3.1 Coping Strategies | Purpose: To enhance FCs’ ability to cope with problems related to their child. Objectives: FCs will be able to correctly apply a five-step problem-solving approach to address practical caregiving problems. | Warm-up and review: Practice self-compassion meditation incorporating guided imagery; invite 1–2 FCs to share experiences related to self-compassion writing. The facilitator guides a discussion on the effects of self-compassion interventions on psychological well-being.3.1.1 Problem-Solving Skills (1) Knowledge-based instruction: A structured problem-solving approach using the IDEAL strategy is introduced. *Identify* the problem: FCs first identify and clearly define the problems encountered in caring for their child. These may involve behavioral issues, communication difficulties, or social skill challenges. *Define* goals: FCs clarify the specific goals they wish to achieve. *Explore solutions*: FCs collaborate with professionals (e.g., physicians, rehabilitation therapists) to explore possible solutions, including behavioral interventions, speech therapy, occupational therapy, or other evidence-based approaches. *Act*: FCs select one solution and commit to implementing it. This may involve specific intervention strategies, such as applied behavior analysis (ABA) techniques or other evidence-based methods. *Look at outcomes*: FCs regularly evaluate the effectiveness of the intervention and adjust strategies as needed. They are encouraged to observe their child’s behavior and progress and to discuss whether modifications are required with professionals.(2) Case analysis: Case 1: A child exhibits restlessness and anxiety while waiting for a medical appointment. Through case analysis, FCs further learn how to apply problem-solving skills in practice. | Lecture  Discussion  Sharing | Each group submits a case report for evaluation, focusing on the comprehensiveness and practicality of the proposed solutions. FCs demonstrate correct application of the five-step problem-solving approach. | Researcher,Rehabilitation therapists | Week 8  90–120 min |
|  |  |  | (3) Group practice: Case 2: The child shows resistance to the noisy environment during a family gathering. Participants are divided into small groups of 3–5 FCs. Each group applies the five-step problem-solving approach to Case 2 to develop a feasible solution. One representative from each group is selected to present the group’s proposed solution. | Group practice  Discussion  Presentation |  |  |  |
| **Note:** The intervention framework and exercises of Mindfulness-based interventions were adapted from the monograph *Mindfulness: An Eight-Week Journey* by Teasdale (2017).  The gratitude intervention combined with expressive writing was informed by the study of Timmons and Ekas (2018).  Acceptance and Commitment Therapy (ACT)–based interventions were informed by and adapted from the book *ACT Made Simple: An Easy-to-Read Primer on Acceptance and Commitment Therapy* (Harris, 2019).  *S*elf-compassion–based interventions were informed by and adapted from the book *Self-Compassion: The Proven Power of Being Kind to Yourself* (Neff, 2011). | | | | | | | |

**References**

1. Teasdale J. 八周正念之旅——摆脱抑郁与情绪压力 [The eight-week mindfulness journey]. Beijing: China Light Industry Press; 2017. Chinese.

2. Timmons L, Ekas NV. Giving thanks: Findings from a gratitude intervention with mothers of children with autism spectrum disorder. Res Autism Spectr Disord. 2018;49: 13–24. doi:10.1016/j.rasd.2018.01.008

3. Harris R. ACT made simple: An easy-to-read primer on acceptance and commitment therapy. 2nd ed. Oakland (CA): New Harbinger Publications; 2019.

4. Neff K. Self-compassion: The proven power of being kind to yourself. New York: William Morrow; 2011.
