# Supplementary material for: An evidence-informed psychosocial intervention program for caregiver burden among family caregivers of children with autism spectrum disorder: a multi-stage development and Delphi study
Source: PLoS One. 2026 Aug 12;21(8):e0354823. doi: 10.1371/journal.pone.0354823 (PMC13465810; doi:10.1371/journal.pone.0354823)
Supplement: Supplementary file 6 [file pone.0354823.s007.docx]

**Response to the Academic Editor and Reviewers**

Manuscript ID: PONE-D-26-16776
An evidence-informed psychosocial intervention program for caregiver burden among family caregivers of children with autism spectrum disorder: a multi-stage development and Delphi study

Dear Academic Editor and Reviewers,

Thank you for the detailed and constructive comments. We revised the manuscript and Supporting Information extensively. Our responses below distinguish changes supported by the study records from reviewer-suggested statements that could not be verified. All substantive changes are included in both a clean manuscript and a tracked-changes manuscript.

# Journal Requirements

## Requirement 1. PLOS ONE style and files

**Comment:** Ensure the revision meets PLOS ONE style and file-naming requirements and provide a response letter, marked manuscript, and clean manuscript.

**Response:** We prepared a point-by-point response, a clean revised manuscript, and a tracked-changes manuscript. The title page, cover letter, and Supporting Information were also revised for consistency.

**Changes in manuscript:** Submission package files; title page; cover letter.

## Requirement 2. Role of funder

**Comment:** State the role of the funder using the requested wording and include it in the cover letter.

**Response:** We added the exact statement requested by the journal: “The funders had no role in study design, data collection and analysis, decision to publish, or preparation of the manuscript.”

**Changes in manuscript:** Revised cover letter and title page, Funding sections.

## Requirement 3. Direct data links

**Comment:** Provide a direct link to each database.

**Response:** To further strengthen compliance with the PLOS data availability policy, we deposited the anonymized Round 1 and Round 2 Delphi consultation datasets, data dictionary, SPSS syntax, and summary analysis outputs in Zenodo: <https://doi.org/10.5281/zenodo.20730110>. Potentially identifiable expert information, including institutional information, contact details, and raw free-text comments, was removed or summarized to protect participant confidentiality. The Data Availability statement has been revised accordingly.

**Changes in manuscript:** Revised title page, Data availability statement; Zenodo data repository.

## Requirement 4. Suggested citations

**Comment:** Evaluate reviewer-suggested publications for relevance; citation is not compulsory unless appropriate.

**Response:** We evaluated each suggested source. ACCORD, the WHO global estimate, the original job demand-control model, and the Delphi review were relevant and were cited. We did not reproduce the proposed global caregiver percentages and daily-hour estimates because a clearly attributable global source and scope could not be verified.

**Changes in manuscript:** Introduction; Study design; Limitations; References.

**Additional compliance update. AI-assisted language-editing disclosure**

**Comment:** PLOS requires contributions by AI tools to the article content to be reported in a dedicated Methods section.

**Response:** We added a dedicated Methods subsection to disclose the limited use of OpenAI ChatGPT during manuscript revision. The tool was used only for English-language editing, consistency checking, and revision-related wording refinement. No AI tool was used to generate or analyse primary data, conduct statistical analyses, create figures or tables, select references, or make scientific interpretations. All AI-assisted text was critically reviewed and revised by the authors, who take full responsibility for the final manuscript.

**Changes in manuscript:** Methods, Use of artificial intelligence tools.

# Academic Editor

## Editor Comment 1. Prior quantitative and qualitative phases

**Comment:** Describe whether the earlier studies were published, who participated, how findings were generated, and how quality and relevance were assessed.

**Response:** We added the designs, dates, settings, sampling, sample sizes, analytic approaches, and quality safeguards for both phases. The quantitative phase included 307 valid surveys from four rehabilitation centres and used regression, SEM, and bootstrap analyses; it is identified as a non-peer-reviewed preprint. The qualitative phase included 16 purposively sampled caregivers, Colaizzi analysis, reflexivity, team analysis, participant feedback, an audit trail, and COREQ reporting. We also explain how relevance and methodological confidence were judged and that no earlier data were re-analysed.

**Changes in manuscript:** Methods, Phase 2: Program conceptualization; Contributions of quantitative and qualitative findings; Discussion.

## Editor Comment 2. Cautious Delphi interpretation

**Comment:** Kendall’s W remained modest; avoid implying strong consensus.

**Response:** We now consistently describe W = 0.23 and 0.25 as statistically significant but modest. Item retention is presented as satisfaction of prespecified item-level criteria, not strong unanimity. Claims of validated feasibility were removed.

**Changes in manuscript:** Abstract Results; Delphi Results; Discussion; Conclusion.

## Editor Comment 3. Caregiver involvement

**Comment:** Explain how caregiver perspectives informed the program or acknowledge that direct caregiver evaluation remains necessary.

**Response:** We clarified that 16 caregiver interviews informed targets, prognosis expectations, delivery preferences, timing, and personnel. We also state explicitly that caregivers did not directly review or co-design the post-Delphi final program and that caregiver focus groups or cognitive interviews are required.

**Changes in manuscript:** Methods, qualitative contribution; Discussion limitations.

## Editor Comment 4. Program feasibility, fatigue, fidelity, and distress

**Comment:** Justify the intensity and explain fatigue/dropout management, fidelity, and emotional-distress procedures.

**Response:** We added the evidence-based rationale for eight weekly sessions and consolidated planned safeguards: hybrid attendance, recordings, small groups, scheduling alongside child rehabilitation, breaks, flexible homework, missed-session follow-up, fatigue monitoring, a standardized manual, facilitator training and rehearsal, checklists, supervision, deviation logs, and a stepped distress-response procedure. These are clearly labelled as plans requiring testing.

**Changes in manuscript:** Results, Implementation planning; Discussion; S4 File overview.

## Editor Comment 5. Moderate feasibility and effectiveness claims

**Comment:** Emphasize intervention development rather than established feasibility or effectiveness.

Response: The Abstract, Discussion, and Conclusion now state that the study developed a structured framework. Acceptability, delivery fidelity, feasibility, and later effectiveness remain to be established.

**Changes in manuscript:** Abstract Conclusions; Discussion; Conclusion.

# Reviewer 1

## Reviewer 1 Comment 1. Ongoing 12-caregiver pilot

**Comment:** Clarify whether preliminary feasibility observations are available or explicitly state they are not included.

**Response:** We state that 12 caregivers had enrolled but no preliminary process, feasibility, or outcome data are included. The pilot will be reported separately after completion.

**Changes in manuscript:** Discussion, Limitations; Supporting Information overview.

## Reviewer 1 Comment 2. Reconciliation of divergent opinions

**Comment:** Explain how divergent expert opinions were reconciled.

**Response:** We added the decision process: ratings and anonymized comments were summarized; recommendations were compared with theory, prior empirical findings, best evidence, clinical feasibility, and internal coherence; decisions were documented; and no item was retained or removed solely because of W.

**Changes in manuscript:** Methods, Delphi procedure and Data analysis.

## Reviewer 1 Comment 3. Implementation and fidelity

**Comment:** Clarify facilitator training, manuals, and consistency across sessions.

**Response:** We added standardized facilitator training, rehearsal and competency checks, a detailed manual, session checklists, attendance and homework records, deviation/adaptation logs, and regular supervision.

**Changes in manuscript:** Results, Implementation planning; S4 File, Intervention personnel and fidelity.

## Reviewer 1 Comment 4. Lengthy Discussion

**Comment:** Condense repetitive Discussion sections.

**Response:** We rewrote the Discussion around four focused issues: evidence integration, multicomponent structure, cautious Delphi interpretation, and implementation/testing implications. Repetitive claims of reliability and feasibility were removed.

**Changes in manuscript:** Discussion.

## Reviewer 1 Comment 5. Terminology

**Comment:** Use family caregivers, caregivers, and FCs consistently.

**Response:** The narrative now uses “family caregivers” at first mention and “caregivers” thereafter. “FCs” is retained mainly in compact tables and detailed Supporting Information.

**Changes in manuscript:** Entire manuscript and Supporting Information.

## Reviewer 1 Comment 6. Language and formatting

**Comment:** Make minor grammatical, stylistic, and formatting revisions.

**Response:** We edited the revised sections for grammar, consistency, cautious causal language, figure callouts, and reference formatting.

**Changes in manuscript:** Entire revised submission.

# Reviewer 2

## Reviewer 2 Comment 1a. Contribution to psychology

**Comment:** Clarify contribution to clinical/health and developmental psychology.

**Response:** The revised theoretical rationale and Discussion explain that the intervention targets caregiver appraisal, psychological resources, stigma, coping, and family interaction, making the framework relevant to health psychology and family/developmental contexts while retaining its nursing and multidisciplinary orientation.

**Changes in manuscript:** Introduction, theoretical rationale; Discussion.

## Reviewer 2 Comment 1b. Global epidemiology and workload

**Comment:** Add updated global epidemiology, including the proposed estimates.

**Response:** We added the verified WHO estimate that approximately 1 in 127 people globally had autism in 2021. We did not present 23–27% psychological-health problems or 13–16 caregiving hours per day as global estimates because the cited review did not provide a clearly verifiable source for those figures. The manuscript instead retains evidence-supported descriptions of substantial and multidimensional burden.

**Changes in manuscript:** Introduction, first paragraph; Response rationale.

## Reviewer 2 Comment 1c. Comparison with the Karasek model

**Comment:** Explain the advantages of the selected theories over the job demand-control-support model.

**Response:** We added a scope-based comparison. The Karasek model was developed for paid work demands, decision latitude, and workplace support. The integrated ABC-X and stress-and-coping framework more directly represents unpaid family caregiving, illness appraisal, family resources, stigma, and coping. We cited the original Karasek model rather than relying on an application in a different occupational context.

**Changes in manuscript:** Introduction, theoretical framework paragraph.

## Reviewer 2 Comment 2a. ACCORD

**Comment:** Note that the study is based on ACCORD.

**Response:** We clarified that the consensus component is reported with reference to ACCORD. ACCORD is a reporting guideline, so we do not state that it was the original basis of a study conducted before the guideline was applied to reporting.

**Changes in manuscript:** Methods, Study design.

## Reviewer 2 Comment 2b. Literature-search start date

**Comment:** The start date is missing.

**Response:** The manuscript already stated that searches covered database inception to September 15, 2024. We retained this wording and confirmed the same period in S1 File.

**Changes in manuscript:** Methods, Literature search; S1 File.

## Reviewer 2 Comment 2c. Figure 1 callout

**Comment:** Insert Figure 1 at the end of the Phase 2 theoretical-basis paragraph.

**Response:** We added “(Fig 1)” at the end of the theoretical-foundation paragraph.

**Changes in manuscript:** Methods, Theoretical foundation of the intervention program.

## Reviewer 2 Comment 3. Data availability and registration

**Comment:** Note the Supporting Information, data availability, and registration ES20245306.

**Response:** We retained registration ES20245306 and the detailed Supporting Information. To further strengthen compliance with the PLOS data availability policy, we deposited the anonymized Round 1 and Round 2 Delphi consultation datasets, data dictionary, SPSS syntax, and summary analysis outputs in Zenodo: <https://doi.org/10.5281/zenodo.20730110>. Potentially identifiable expert information, including institutional information, contact details, and raw free-text comments, was removed or summarized to protect participant confidentiality. The Data Availability statement has been revised accordingly.

**Changes in manuscript:** Revised title page, Data availability statement; S1–S4 Files; Zenodo data repository.

## Reviewer 2 Comment 4a. Citation for fragmented approaches

**Comment:** Cite support for the claim that current approaches are fragmented.

**Response:** We retained and clarified support from the existing systematic reviews and meta-analyses. We also moderated the statement so that heterogeneity and fragmentation do not become an unsupported claim that the new multicomponent package is superior.

**Changes in manuscript:** Introduction; Discussion, second paragraph.

## Reviewer 2 Comment 4b. Delphi format limitation

**Comment:** State that the Delphi was one phase with two asynchronous rounds and compare it with a proposed three-phase, three-round synchronous approach.

**Response:** We now identify the process as a two-round asynchronous modified Delphi with summarized feedback and acknowledge the lack of synchronous deliberation. We did not state that a “complete Delphi” universally requires three phases, three rounds, or in-person consultation because Delphi methods vary and the cited narrative review does not establish a universal requirement. This methodological nuance is now explicit.

**Changes in manuscript:** Methods, Delphi procedure; Discussion, Limitations.

## Reviewer 2 Comment 4c. Caregiver focus groups

**Comment:** The program should ideally be validated through caregiver focus groups.

**Response:** We agree. We added caregiver focus groups or cognitive interviews as the next required step to assess acceptability, cultural fit, participation burden, emotional safety, and practical feasibility before a definitive trial.

**Changes in manuscript:** Discussion, Limitations and Conclusion.
