# Supplementary material for: An evidence-informed psychosocial intervention program for caregiver burden among family caregivers of children with autism spectrum disorder: a multi-stage development and Delphi study
Source: PLoS One. 2026 Aug 12;21(8):e0354823. doi: 10.1371/journal.pone.0354823 (PMC13465810; doi:10.1371/journal.pone.0354823)
Supplement: Supplementary file 7 [file pone.0354823.s008.docx]

**Response to Reviewers**

**Manuscript ID:** PONE-D-26-16776R1
**Title:** An evidence-informed psychosocial intervention program for caregiver burden among family caregivers of children with autism spectrum disorder: a multi-stage development and Delphi study

Dear Dr. Jiang and Reviewer,

We sincerely thank the Academic Editor and Reviewer #2 for the careful evaluation of our revised manuscript and for the constructive comments that have helped us further clarify the scope and contribution of the study. We are grateful that Reviewer #2 considered the previous comments to have been adequately addressed. In this revision, we have focused on the remaining conceptual issue raised by the Academic Editor regarding the interpretation of the Delphi findings and the distinction between program development and formal validation or demonstrated feasibility/effectiveness.

All changes have been made in the revised manuscript and are shown in the tracked-changes version. A clean version of the manuscript is also provided.

**Response to Academic Editor**

**Comment 1**

The revised manuscript is substantially improved and now presents a clearer and more appropriately cautious account of the intervention-development process. The authors have responded carefully to the concerns raised about the earlier quantitative and qualitative phases, the interpretation of the Delphi findings, caregiver involvement, implementation planning, and the distinction between program development and demonstrated feasibility or effectiveness.

I particularly appreciate that the revised manuscript now describes Kendall’s W as modest, acknowledges that caregivers did not directly review the final post-Delphi program, and clearly states that the ongoing pilot data are not included in the present paper. These revisions make the scope and contribution of the study much more transparent.

**Response:**
We sincerely thank the Academic Editor for this positive and constructive assessment. We appreciate the recognition that the revised manuscript now presents a clearer and more cautious account of the intervention-development process, including the modest Kendall’s W values, the indirect nature of caregiver involvement, and the distinction between the present development study and ongoing pilot work. These points have been carefully retained in the current revision.

**Comment 2**

I have only one remaining conceptual concern. In several places, the manuscript still appears to suggest that the evidence synthesis and Delphi process support the “reliability” or “content validity” of the intervention program. These terms may be stronger than the present design allows. The study demonstrates systematic development, expert appraisal, and refinement of the program, but it does not appear to include a formal reliability assessment or a conventional content-validity analysis. I therefore suggest replacing “reliability” with wording such as “systematic refinement and expert appraisal,” and replacing “content validity” with “perceived relevance, clarity, and appropriateness,” unless formal content-validity indices were calculated.

**Response:**
Thank you for this important clarification. We fully agree that the present study did not include a formal reliability assessment or a conventional content-validity analysis, and that the evidence synthesis and Delphi process should not be interpreted as establishing the reliability or formal content validity of the intervention program.

In response, we carefully reviewed the manuscript and revised wording that could imply stronger methodological claims than the design supports. We now describe the contribution of the evidence synthesis and Delphi consultation as **systematic development, expert appraisal, and refinement of the program**, rather than as evidence of reliability or formal validity.

Specifically, we revised the relevant wording in the Abstract, Discussion, and Conclusion. The manuscript now refers to the Delphi findings as reflecting experts’ **perceptions of the relevance, clarity, and appropriateness** of the program content, rather than as establishing formal content validity.

For example, the Results section of the Abstract has been revised to state that the Delphi process contributed to systematic refinement and expert appraisal of the program and reflected experts’ perceptions of the relevance, clarity, and appropriateness of its content, while practical feasibility remains to be tested directly with caregivers.

In the Discussion, we revised the interpretation of the Delphi findings to clarify that the ratings should be understood as expert perceptions rather than evidence of formal content validity, strong consensus, feasibility, or effectiveness.

**Comment 3**

It would also be helpful to ensure that the Conclusion continues to maintain the same cautious distinction used elsewhere in the manuscript: the program is a structured and evidence-informed framework ready for caregiver-informed feasibility testing, rather than an intervention whose feasibility or effectiveness has already been established.

**Response:**
We agree and have revised the Conclusion to maintain this cautious distinction more explicitly. The revised Conclusion now states that the program is a structured, theory-driven, and evidence-informed framework developed for subsequent caregiver-informed feasibility testing. We also explicitly state that the Delphi process enabled systematic refinement and expert appraisal, but did not establish formal content validity, feasibility, or effectiveness.

The revised Conclusion further emphasizes that the framework is ready for direct caregiver-informed feasibility testing, including assessment of acceptability, delivery fidelity, participant burden, cultural fit, and emotional safety, before subsequent effectiveness evaluation.

**Response to Reviewer #2**

**Comment**

All comments have been addressed.

**Response:**
We sincerely thank Reviewer #2 for confirming that the previous comments have been adequately addressed and for the positive assessment of the revised manuscript. We also appreciate the reviewer’s recognition of the inclusion of the ACCORD guideline. No additional revisions were requested by Reviewer #2 in this round.

We thank the Academic Editor and Reviewer again for their time and constructive feedback. We hope that the revised manuscript now adequately addresses the remaining concerns and meets the publication criteria of PLOS ONE.

Sincerely,
Xiuchuan Yan, PhD
On behalf of all authors
