# Supplementary material for: An evidence-informed psychosocial intervention program for caregiver burden among family caregivers of children with autism spectrum disorder: a multi-stage development and Delphi study
Source: PLoS One. 2026 Aug 12;21(8):e0354823. doi: 10.1371/journal.pone.0354823 (PMC13465810; doi:10.1371/journal.pone.0354823)
Supplement: Peer review history

# Peer review: Decision Letter 0

**DOI:** 10.1371/journal.pone.0354823.r001

**Authors:** Yilin Jiang

28 May 2026

PONE-D-26-16776

An evidence-informed psychosocial intervention program for caregiver burden among family caregivers of children with autism spectrum disorder: a multi-stage development and Delphi study

PLOS One

Dear Dr. Yan,

Thank you for submitting your manuscript to PLOS ONE. After careful consideration, we feel that it has merit but does not fully meet PLOS ONE’s publication criteria as it currently stands. Therefore, we invite you to submit a revised version of the manuscript that addresses the points raised during the review process.

Please submit your revised manuscript by Jul 12 2026 11:59PM. If you will need more time than this to complete your revisions, please reply to this message or contact the journal office at plosone@plos.org. When you're ready to submit your revision, log on to https://www.editorialmanager.com/pone/ and select the 'Submissions Needing Revision' folder to locate your manuscript file.

Please include the following items when submitting your revised manuscript:

- A letter that responds to each point raised by the academic editor and reviewer(s). You should upload this letter as a separate file labeled 'Response to Reviewers'.
- A marked-up copy of your manuscript that highlights changes made to the original version. You should upload this as a separate file labeled 'Revised Manuscript with Track Changes'.
- An unmarked version of your revised paper without tracked changes. You should upload this as a separate file labeled 'Manuscript'.

If you would like to make changes to your financial disclosure, please include your updated statement in your cover letter. Guidelines for resubmitting your figure files are available below the reviewer comments at the end of this letter.

If applicable, we recommend that you deposit your laboratory protocols in protocols.io to enhance the reproducibility of your results. Protocols.io assigns your protocol its own identifier (DOI) so that it can be cited independently in the future. For instructions see: https://journals.plos.org/plosone/s/submission-guidelines#loc-laboratory-protocols. Additionally, PLOS ONE offers an option for publishing peer-reviewed Lab Protocol articles, which describe protocols hosted on protocols.io. Read more information on sharing protocols at https://plos.org/protocols?utm_medium=editorial-email&utm_source=authorletters&utm_campaign=protocols.

As the corresponding author, your ORCID iD is verified in the submission system and will appear in the published article. PLOS supports the use of ORCID, and we encourage all coauthors to register for an ORCID iD and use it as well. Please encourage your coauthors to verify their ORCID iD within the submission system before final acceptance, as unverified ORCID iDs will not appear in the published article. *Only*  the individual author can complete the verification step; PLOS staff *cannot*  verify ORCID iDs on behalf of authors.

We look forward to receiving your revised manuscript.

Kind regards,

Yilin Jiang

Academic Editor

PLOS One

Journal Requirements:

When submitting your revision, we need you to address these additional requirements.

1. Please ensure that your manuscript meets PLOS ONE's style requirements, including those for file naming. The PLOS ONE style templates can be found at

https://journals.plos.org/plosone/s/file?id=wjVg/PLOSOne_formatting_sample_main_body.pdf and

https://journals.plos.org/plosone/s/file?id=ba62/PLOSOne_formatting_sample_title_authors_affiliations.pdf

2. Thank you for stating the following financial disclosure:

“Shenzhen Longgang District Science and Technology Innovation Special Fund—Medical and Health Technology Project(Grant No. LGWJ2023-098.”

Please state what role the funders took in the study.  If the funders had no role, please state: "The funders had no role in study design, data collection and analysis, decision to publish, or preparation of the manuscript."

If this statement is not correct you must amend it as needed.

Please include this amended Role of Funder statement in your cover letter; we will change the online submission form on your behalf.

3.  Please note that your Data Availability Statement is currently missing a direct link to access each database. If your manuscript is accepted for publication, you will be asked to provide these details on a very short timeline. We therefore suggest that you provide this information now, though we will not hold up the peer review process if you are unable.

4. If the reviewer comments include a recommendation to cite specific previously published works, please review and evaluate these publications to determine whether they are relevant and should be cited. There is no requirement to cite these works unless the editor has indicated otherwise.

Additional Editor Comments:

First, the role of the prior quantitative and qualitative phases needs to be described more transparently. These previous findings appear to be central to the development of the intervention targets, such as psychological capital, perceived social support, illness cognition, affiliate stigma, and coping strategies. However, the manuscript does not provide enough detail about these earlier phases. Please clarify whether these studies have been published, who the participants were, how the findings were generated, and how their quality and relevance were assessed. Since these earlier phases directly shaped the intervention program, readers need more information to judge the strength of this evidence base.

Second, the Delphi results should be interpreted more cautiously. The expert response rate was high, and the authority coefficient was acceptable, which supports the credibility of the consultation process. However, Kendall’s W remained modest across the two rounds, increasing only slightly from 0.23 to 0.25. This suggests statistically significant but limited agreement among experts. Therefore, the manuscript should avoid implying strong expert consensus. It would be more accurate to state that the Delphi process provided preliminary support for content validity and feasibility, rather than strong confirmation of the program’s validity.

Third, the manuscript should address the limited involvement of caregiver stakeholders. The intervention is designed for family caregivers of children with ASD, but the final refinement appears to rely mainly on professional experts. Expert input is valuable, but caregiver acceptability, burden, cultural fit, and practical feasibility cannot be fully assessed without caregiver feedback. Please either explain how caregiver perspectives from the prior qualitative phase informed the final program, or acknowledge more clearly that direct caregiver evaluation remains necessary in future feasibility testing.

Fourth, the feasibility of the final program needs stronger justification. The finalized intervention includes three modules, seven themes, 22 components, and eight sessions lasting 90–120 minutes each. It also combines multiple approaches, including mindfulness, gratitude-based expressive writing, ACT-informed strategies, self-compassion training, and problem-solving skills. This is a rich program, but it may also be demanding for caregivers who already face substantial time, emotional, and practical burdens. The authors should explain why this intensity is feasible, how participant fatigue or dropout will be managed, how intervention fidelity will be maintained, and how emotional distress during sessions will be handled.

Finally, I encourage the authors to moderate claims about feasibility and effectiveness. Since pilot feasibility testing is still ongoing and effectiveness has not yet been evaluated, the conclusion should emphasize that this study developed a structured and evidence-informed intervention framework, rather than suggesting that the program is already feasible or effective in practice.

Overall, this is a promising and relevant manuscript, but it requires substantial clarification and refinement before publication. The study would be strengthened by greater transparency about the evidence sources, more cautious interpretation of the Delphi findings, clearer acknowledgement of caregiver involvement, and stronger justification of the program’s practical feasibility.

Reviewers' comments:

Reviewer's Responses to Questions

**Comments to the Author**

1. Is the manuscript technically sound, and do the data support the conclusions?

The manuscript must describe a technically sound piece of scientific research with data that supports the conclusions. Experiments must have been conducted rigorously, with appropriate controls, replication, and sample sizes. The conclusions must be drawn appropriately based on the data presented.

Reviewer #1: Yes

Reviewer #2: Yes

**********

2. Has the statistical analysis been performed appropriately and rigorously?

Reviewer #1: Yes

Reviewer #2: Yes

**********

3. Have the authors made all data underlying the findings in their manuscript fully available?

The PLOS Data policy requires authors to make all data underlying the findings described in their manuscript fully available without restriction, with rare exception (please refer to the Data Availability Statement in the manuscript PDF file). The data should be provided as part of the manuscript or its supporting information, or deposited to a public repository. For example, in addition to summary statistics, the data points behind means, medians and variance measures should be available. If there are restrictions on publicly sharing data—e.g. participant privacy or use of data from a third party—those must be specified.

Reviewer #1: Yes

Reviewer #2: Yes

**********

4. Is the manuscript presented in an intelligible fashion and written in standard English?

PLOS ONE does not copyedit accepted manuscripts, so the language in submitted articles must be clear, correct, and unambiguous. Any typographical or grammatical errors should be corrected at revision, so please note any specific errors here.

Reviewer #1: Yes

Reviewer #2: Yes

**********

5. Review Comments to the Author

Please use the space provided to explain your answers to the questions above. You may also include additional comments for the author, including concerns about dual publication, research ethics, or publication ethics. (Please upload your review as an attachment if it exceeds 20,000 characters)

Reviewer #1: This manuscript presents a well-structured, theory-informed, and evidence-based psychosocial intervention program targeting caregiver burden among family caregivers of children with autism spectrum disorder (ASD). The topic is important and clinically relevant, particularly given the growing recognition of caregiver burden and the limited availability of structured caregiver-focused psychosocial interventions in this population. The manuscript demonstrates a thoughtful and rigorous multi-stage intervention development process integrating theoretical frameworks, evidence synthesis, prior quantitative and qualitative findings, and Delphi expert consultation.

The methodological approach is generally sound and clearly described. The integration of the ABC-X family stress model and stress and coping theory provides a coherent conceptual foundation for the intervention structure. The evidence synthesis process was systematic and appropriately conducted, and the Delphi consultation included multidisciplinary experts with substantial professional experience. The statistical analyses used for the Delphi process, including Kendall’s coefficient of concordance, coefficients of variation, and expert authority coefficients, were appropriate for this type of methodological study.

The manuscript is generally well written and organized, and the conclusions are appropriately cautious and aligned with the developmental nature of the study. Importantly, the authors appropriately acknowledge that intervention feasibility and effectiveness have not yet been fully evaluated.

I have several suggestions that may further strengthen the manuscript:

The manuscript mentions that pilot feasibility testing has already been initiated with 12 family caregivers enrolled. Please clarify whether any preliminary feasibility observations or process indicators are currently available, or explicitly state that no feasibility outcomes are included in the present study.

Although the Delphi consensus achieved statistical significance, Kendall’s W values remained modest. The discussion addresses this appropriately; however, additional clarification regarding how divergent expert opinions were reconciled during the revision process would improve transparency.

More detail regarding intervention implementation and fidelity management would strengthen the practical applicability of the program. For example, please clarify whether facilitators will receive standardized training, whether intervention manuals will be used, and how consistency across sessions will be maintained.

Some sections of the Discussion are relatively lengthy and repetitive. Condensing several paragraphs may improve readability and manuscript flow.

Please ensure consistency in terminology throughout the manuscript, particularly regarding the use of “family caregivers,” “caregivers,” and “FCs.”

Minor grammatical, stylistic, and formatting revisions are recommended to improve clarity and readability.

Overall, this is a valuable and meaningful contribution to the literature on psychosocial support for caregivers of children with ASD. The study provides an important foundation for future feasibility and effectiveness research, and the intervention framework may have practical relevance across pediatric, rehabilitation, nursing, and community care settings. After minor revision, the manuscript would be suitable for publication.

Reviewer #2: The authors propose a multidisciplinary intervention program that will contribute significantly to both the Sustainable Development Goals (SDGs: 3, 4, 5) and the fields of health and behavioral sciences. A strength of the study is the evaluation of the quality of evidence and the components of the intervention program.

1. Statements of interest and the authors’ position on the knowledge gap

Introduction

The authors justify the need to develop a multidisciplinary intervention based on a Rapid Review, three scientific theories, and expert consultation. They also highlight the need to adopt an intervention that goes beyond merely reducing symptoms, opting instead for a structured, multi-component, multidisciplinary psychosocial intervention, as recommended by the WHO (World Health Organization). They also discuss the epidemiology of ASD (Autism Spectrum Disorder) in children, though without providing global figures to support their claims. Therefore, it is appropriate for the authors to note the following:

a) The program’s specific contribution, in addition to medical sciences, to clinical and health psychology, and to developmental psychology (because the program will improve the three-way interaction between caregivers, children, and family members).

b) The updated global epidemiology regarding the workload of caregivers of children with ASD. In this regard, it is known that approximately one in every 127 children has ASD, and between 23–27% of these children’s caregivers experience psychological health problems with a workload of up to 13–16 hours per day.

Highlight the advantages of using the scientific theories presented in Figure 1 compared to the “Karasek Theoretical Model of Demand-Control-Psychosocial Support in the Work Environment,” an analysis of the theoretical perspective of which can be found on pages 8 and 9 of: https://www.medwave.cl/medios//research/studies/2986/medwave_2024_2986-1.pdf

2. Details of the methods and techniques used

Methods

They are sufficient. However, after review, it should be noted that the study is based on the ACCORD (ACcurate COnsensus Reporting Document) guideline from the EQUATOR Network published by Gattrell et al. (2024) in PLOS Med, 21.

Literature search: the end date is indicated, but the start date is missing.

At the end of the paragraph “Phase 2. Theoretical basis… insert (Figure 1).

2. Data support and analysis techniques

Sufficient.

3. Data availability

In addition to sufficient data availability as supplementary material and deposited in “Zenodo,” the evidence synthesis is also registered (ES20245306).

4. Ethical considerations

Adequate. The research project was approved by a Research Ethics Committee (KY-2024-368).

Discussion

The authors state “...the most fragmented intervention approaches when combining multiple components within a single structured framework...”; please cite the references supporting this claim (2nd paragraph).

As limitations:

a) Note that the Delphi method was conducted in a single phase consisting of two rounds of asynchronous consultations (the complete Delphi method comprises three phases with three rounds of in-person or synchronous consultations in the second phase, i.e., controlled and with feedback (Shang, 2023: https://doi.org/10. 1097/MD.0000000000032829).

b) Ideally, the intervention program should be validated through focus groups (caregivers of children with ASD).

**********

6. PLOS authors have the option to publish the peer review history of their article (what does this mean?). If published, this will include your full peer review and any attached files.

If you choose “no”, your identity will remain anonymous but your review may still be made public.

**Do you want your identity to be public for this peer review?**  For information about this choice, including consent withdrawal, please see our Privacy Policy.

Reviewer #1: No

Reviewer #2: No

**********

[NOTE: If reviewer comments were submitted as an attachment file, they will be attached to this email and accessible via the submission site. Please log into your account, locate the manuscript record, and check for the action link "View Attachments". If this link does not appear, there are no attachment files.]

To ensure your figures meet our technical requirements, please review our figure guidelines: https://journals.plos.org/plosone/s/figures

You may also use PLOS’s free figure tool, NAAS, to help you prepare publication quality figures: https://journals.plos.org/plosone/s/figures#loc-tools-for-figure-preparation.

NAAS will assess whether your figures meet our technical requirements by comparing each figure against our figure specifications.

Attachment Submitted filename: REVIEW REPORT_PONE-D-26-16776 en-US.pdf

# Author comment: Author response to Decision Letter 1

**DOI:** 10.1371/journal.pone.0354823.r002

20 Jun 2026

Response to the Academic Editor and Reviewers

Manuscript ID: PONE-D-26-16776

An evidence-informed psychosocial intervention program for caregiver burden among family caregivers of children with autism spectrum disorder: a multi-stage development and Delphi study

Dear Academic Editor and Reviewers,

Thank you for the detailed and constructive comments. We revised the manuscript and Supporting Information extensively. Our responses below distinguish changes supported by the study records from reviewer-suggested statements that could not be verified. All substantive changes are included in both a clean manuscript and a tracked-changes manuscript.

Journal Requirements

Requirement 1. PLOS ONE style and files

Comment: Ensure the revision meets PLOS ONE style and file-naming requirements and provide a response letter, marked manuscript, and clean manuscript.

Response: We prepared a point-by-point response, a clean revised manuscript, and a tracked-changes manuscript. The title page, cover letter, and Supporting Information were also revised for consistency.

Changes in manuscript: Submission package files; title page; cover letter.

Requirement 2. Role of funder

Comment: State the role of the funder using the requested wording and include it in the cover letter.

Response: We added the exact statement requested by the journal: “The funders had no role in study design, data collection and analysis, decision to publish, or preparation of the manuscript.”

Changes in manuscript: Revised cover letter and title page, Funding sections.

Requirement 3. Direct data links

Comment: Provide a direct link to each database.

Response: To further strengthen compliance with the PLOS data availability policy, we deposited the anonymized Round 1 and Round 2 Delphi consultation datasets, data dictionary, SPSS syntax, and summary analysis outputs in Zenodo: https://doi.org/10.5281/zenodo.20730110. Potentially identifiable expert information, including institutional information, contact details, and raw free-text comments, was removed or summarized to protect participant confidentiality. The Data Availability statement has been revised accordingly.

Changes in manuscript: Revised title page, Data availability statement; Zenodo data repository.

Requirement 4. Suggested citations

Comment: Evaluate reviewer-suggested publications for relevance; citation is not compulsory unless appropriate.

Response: We evaluated each suggested source. ACCORD, the WHO global estimate, the original job demand-control model, and the Delphi review were relevant and were cited. We did not reproduce the proposed global caregiver percentages and daily-hour estimates because a clearly attributable global source and scope could not be verified.

Changes in manuscript: Introduction; Study design; Limitations; References.

Additional compliance update. AI-assisted language-editing disclosure

Comment: PLOS requires contributions by AI tools to the article content to be reported in a dedicated Methods section.

Response: We added a dedicated Methods subsection to disclose the limited use of OpenAI ChatGPT during manuscript revision. The tool was used only for English-language editing, consistency checking, and revision-related wording refinement. No AI tool was used to generate or analyse primary data, conduct statistical analyses, create figures or tables, select references, or make scientific interpretations. All AI-assisted text was critically reviewed and revised by the authors, who take full responsibility for the final manuscript.

Changes in manuscript: Methods, Use of artificial intelligence tools.

Academic Editor

Editor Comment 1. Prior quantitative and qualitative phases

Comment: Describe whether the earlier studies were published, who participated, how findings were generated, and how quality and relevance were assessed.

Response: We added the designs, dates, settings, sampling, sample sizes, analytic approaches, and quality safeguards for both phases. The quantitative phase included 307 valid surveys from four rehabilitation centres and used regression, SEM, and bootstrap analyses; it is identified as a non-peer-reviewed preprint. The qualitative phase included 16 purposively sampled caregivers, Colaizzi analysis, reflexivity, team analysis, participant feedback, an audit trail, and COREQ reporting. We also explain how relevance and methodological confidence were judged and that no earlier data were re-analysed.

Changes in manuscript: Methods, Phase 2: Program conceptualization; Contributions of quantitative and qualitative findings; Discussion.

Editor Comment 2. Cautious Delphi interpretation

Comment: Kendall’s W remained modest; avoid implying strong consensus.

Response: We now consistently describe W = 0.23 and 0.25 as statistically significant but modest. Item retention is presented as satisfaction of prespecified item-level criteria, not strong unanimity. Claims of validated feasibility were removed.

Changes in manuscript: Abstract Results; Delphi Results; Discussion; Conclusion.

Editor Comment 3. Caregiver involvement

Comment: Explain how caregiver perspectives informed the program or acknowledge that direct caregiver evaluation remains necessary.

Response: We clarified that 16 caregiver interviews informed targets, prognosis expectations, delivery preferences, timing, and personnel. We also state explicitly that caregivers did not directly review or co-design the post-Delphi final program and that caregiver focus groups or cognitive interviews are required.

Changes in manuscript: Methods, qualitative contribution; Discussion limitations.

Editor Comment 4. Program feasibility, fatigue, fidelity, and distress

Comment: Justify the intensity and explain fatigue/dropout management, fidelity, and emotional-distress procedures.

Response: We added the evidence-based rationale for eight weekly sessions and consolidated planned safeguards: hybrid attendance, recordings, small groups, scheduling alongside child rehabilitation, breaks, flexible homework, missed-session follow-up, fatigue monitoring, a standardized manual, facilitator training and rehearsal, checklists, supervision, deviation logs, and a stepped distress-response procedure. These are clearly labelled as plans requiring testing.

Changes in manuscript: Results, Implementation planning; Discussion; S4 File overview.

Editor Comment 5. Moderate feasibility and effectiveness claims

Comment: Emphasize intervention development rather than established feasibility or effectiveness.

Response: The Abstract, Discussion, and Conclusion now state that the study developed a structured framework. Acceptability, delivery fidelity, feasibility, and later effectiveness remain to be established.

Changes in manuscript: Abstract Conclusions; Discussion; Conclusion.

Reviewer 1

Reviewer 1 Comment 1. Ongoing 12-caregiver pilot

Comment: Clarify whether preliminary feasibility observations are available or explicitly state they are not included.

Response: We state that 12 caregivers had enrolled but no preliminary process, feasibility, or outcome data are included. The pilot will be reported separately after completion.

Changes in manuscript: Discussion, Limitations; Supporting Information overview.

Reviewer 1 Comment 2. Reconciliation of divergent opinions

Comment: Explain how divergent expert opinions were reconciled.

Response: We added the decision process: ratings and anonymized comments were summarized; recommendations were compared with theory, prior empirical findings, best evidence, clinical feasibility, and internal coherence; decisions were documented; and no item was retained or removed solely because of W.

Changes in manuscript: Methods, Delphi procedure and Data analysis.

Reviewer 1 Comment 3. Implementation and fidelity

Comment: Clarify facilitator training, manuals, and consistency across sessions.

Response: We added standardized facilitator training, rehearsal and competency checks, a detailed manual, session checklists, attendance and homework records, deviation/adaptation logs, and regular supervision.

Changes in manuscript: Results, Implementation planning; S4 File, Intervention personnel and fidelity.

Reviewer 1 Comment 4. Lengthy Discussion

Comment: Condense repetitive Discussion sections.

Response: We rewrote the Discussion around four focused issues: evidence integration, multicomponent structure, cautious Delphi interpretation, and implementation/testing implications. Repetitive claims of reliability and feasibility were removed.

Changes in manuscript: Discussion.

Reviewer 1 Comment 5. Terminology

Comment: Use family caregivers, caregivers, and FCs consistently.

Response: The narrative now uses “family caregivers” at first mention and “caregivers” thereafter. “FCs” is retained mainly in compact tables and detailed Supporting Information.

Changes in manuscript: Entire manuscript and Supporting Information.

Reviewer 1 Comment 6. Language and formatting

Comment: Make minor grammatical, stylistic, and formatting revisions.

Response: We edited the revised sections for grammar, consistency, cautious causal language, figure callouts, and reference formatting.

Changes in manuscript: Entire revised submission.

Reviewer 2

Reviewer 2 Comment 1a. Contribution to psychology

Comment: Clarify contribution to clinical/health and developmental psychology.

Response: The revised theoretical rationale and Discussion explain that the intervention targets caregiver appraisal, psychological resources, stigma, coping, and family interaction, making the framework relevant to health psychology and family/developmental contexts while retaining its nursing and multidisciplinary orientation.

Changes in manuscript: Introduction, theoretical rationale; Discussion.

Reviewer 2 Comment 1b. Global epidemiology and workload

Comment: Add updated global epidemiology, including the proposed estimates.

Response: We added the verified WHO estimate that approximately 1 in 127 people globally had autism in 2021. We did not present 23–27% psychological-health problems or 13–16 caregiving hours per day as global estimates because the cited review did not provide a clearly verifiable source for those figures. The manuscript instead retains evidence-supported descriptions of substantial and multidimensional burden.

Changes in manuscript: Introduction, first paragraph; Response rationale.

Reviewer 2 Comment 1c. Comparison with the Karasek model

Comment: Explain the advantages of the selected theories over the job demand-control-support model.

Response: We added a scope-based comparison. The Karasek model was developed for paid work demands, decision latitude, and workplace support. The integrated ABC-X and stress-and-coping framework more directly represents unpaid family caregiving, illness appraisal, family resources, stigma, and coping. We cited the original Karasek model rather than relying on an application in a different occupational context.

Changes in manuscript: Introduction, theoretical framework paragraph.

Reviewer 2 Comment 2a. ACCORD

Comment: Note that the study is based on ACCORD.

Response: We clarified that the consensus component is reported with reference to ACCORD. ACCORD is a reporting guideline, so we do not state that it was the original basis of a study conducted before the guideline was applied to reporting.

Changes in manuscript: Methods, Study design.

Reviewer 2 Comment 2b. Literature-search start date

Comment: The start date is missing.

Response: The manuscript already stated that searches covered database inception to September 15, 2024. We retained this wording and confirmed the same period in S1 File.

Changes in manuscript: Methods, Literature search; S1 File.

Reviewer 2 Comment 2c. Figure 1 callout

Comment: Insert Figure 1 at the end of the Phase 2 theoretical-basis paragraph.

Response: We added “(Fig 1)” at the end of the theoretical-foundation paragraph.

Changes in manuscript: Methods, Theoretical foundation of the intervention program.

Reviewer 2 Comment 3. Data availability and registration

Comment: Note the Supporting Information, data availability, and registration ES20245306.

Response: We retained registration ES20245306 and the detailed Supporting Information. To further strengthen compliance with the PLOS data availability policy, we deposited the anonymized Round 1 and Round 2 Delphi consultation datasets, data dictionary, SPSS syntax, and summary analysis outputs in Zenodo: https://doi.org/10.5281/zenodo.20730110. Potentially identifiable expert information, including institutional information, contact details, and raw free-text comments, was removed or summarized to protect participant confidentiality. The Data Availability statement has been revised accordingly.

Changes in manuscript: Revised title page, Data availability statement; S1–S4 Files; Zenodo data repository.

Reviewer 2 Comment 4a. Citation for fragmented approaches

Comment: Cite support for the claim that current approaches are fragmented.

Response: We retained and clarified support from the existing systematic reviews and meta-analyses. We also moderated the statement so that heterogeneity and fragmentation do not become an unsupported claim that the new multicomponent package is superior.

Changes in manuscript: Introduction; Discussion, second paragraph.

Reviewer 2 Comment 4b. Delphi format limitation

Comment: State that the Delphi was one phase with two asynchronous rounds and compare it with a proposed three-phase, three-round synchronous approach.

Response: We now identify the process as a two-round asynchronous modified Delphi with summarized feedback and acknowledge the lack of synchronous deliberation. We did not state that a “complete Delphi” universally requires three phases, three rounds, or in-person consultation because Delphi methods vary and the cited narrative review does not establish a universal requirement. This methodological nuance is now explicit.

Changes in manuscript: Methods, Delphi procedure; Discussion, Limitations.

Reviewer 2 Comment 4c. Caregiver focus groups

Comment: The program should ideally be validated through caregiver focus groups.

Response: We agree. We added caregiver focus groups or cognitive interviews as the next required step to assess acceptability, cultural fit, participation burden, emotional safety, and practical feasibility before a definitive trial.

Changes in manuscript: Discussion, Limitations and Conclusion.

Attachment Submitted filename: Response to Reviewers.docx

# Peer review: Decision Letter 1

**DOI:** 10.1371/journal.pone.0354823.r003

**Authors:** Yilin Jiang

8 Jul 2026

PONE-D-26-16776R1

An evidence-informed psychosocial intervention program for caregiver burden among family caregivers of children with autism spectrum disorder: a multi-stage development and Delphi study

PLOS One

Dear Dr. Yan,

Thank you for submitting your manuscript to PLOS ONE. After careful consideration, we feel that it has merit but does not fully meet PLOS ONE’s publication criteria as it currently stands. Therefore, we invite you to submit a revised version of the manuscript that addresses the points raised during the review process.

Please submit your revised manuscript by Aug 22 2026 11:59PM. If you will need more time than this to complete your revisions, please reply to this message or contact the journal office at plosone@plos.org. When you're ready to submit your revision, log on to https://www.editorialmanager.com/pone/ and select the 'Submissions Needing Revision' folder to locate your manuscript file.

Please include the following items when submitting your revised manuscript:

- A letter that responds to each point raised by the academic editor and reviewer(s). You should upload this letter as a separate file labeled 'Response to Reviewers'.
- A marked-up copy of your manuscript that highlights changes made to the original version. You should upload this as a separate file labeled 'Revised Manuscript with Track Changes'.
- An unmarked version of your revised paper without tracked changes. You should upload this as a separate file labeled 'Manuscript'.

If you would like to make changes to your financial disclosure, please include your updated statement in your cover letter. Guidelines for resubmitting your figure files are available below the reviewer comments at the end of this letter.

If applicable, we recommend that you deposit your laboratory protocols in protocols.io to enhance the reproducibility of your results. Protocols.io assigns your protocol its own identifier (DOI) so that it can be cited independently in the future. For instructions see: https://journals.plos.org/plosone/s/submission-guidelines#loc-laboratory-protocols. Additionally, PLOS ONE offers an option for publishing peer-reviewed Lab Protocol articles, which describe protocols hosted on protocols.io. Read more information on sharing protocols at https://plos.org/protocols?utm_medium=editorial-email&utm_source=authorletters&utm_campaign=protocols.

As the corresponding author, your ORCID iD is verified in the submission system and will appear in the published article. PLOS supports the use of ORCID, and we encourage all coauthors to register for an ORCID iD and use it as well. Please encourage your coauthors to verify their ORCID iD within the submission system before final acceptance, as unverified ORCID iDs will not appear in the published article. *Only*  the individual author can complete the verification step; PLOS staff *cannot*  verify ORCID iDs on behalf of authors.

We look forward to receiving your revised manuscript.

Kind regards,

Yilin Jiang

Academic Editor

PLOS One

Journal Requirements:

If the reviewer comments include a recommendation to cite specific previously published works, please review and evaluate these publications to determine whether they are relevant and should be cited. There is no requirement to cite these works unless the editor has indicated otherwise.

Please review your reference list to ensure that it is complete and correct. If you have cited papers that have been retracted, please include the rationale for doing so in the manuscript text, or remove these references and replace them with relevant current references. Any changes to the reference list should be mentioned in the rebuttal letter that accompanies your revised manuscript. If you need to cite a retracted article, indicate the article’s retracted status in the References list and also include a citation and full reference for the retraction notice.

Additional Editor Comments:

The revised manuscript is substantially improved and now presents a clearer and more appropriately cautious account of the intervention-development process. The authors have responded carefully to the concerns raised about the earlier quantitative and qualitative phases, the interpretation of the Delphi findings, caregiver involvement, implementation planning, and the distinction between program development and demonstrated feasibility or effectiveness.

I particularly appreciate that the revised manuscript now describes Kendall’s W as modest, acknowledges that caregivers did not directly review the final post-Delphi program, and clearly states that the ongoing pilot data are not included in the present paper. These revisions make the scope and contribution of the study much more transparent.

I have only one remaining conceptual concern. In several places, the manuscript still appears to suggest that the evidence synthesis and Delphi process support the “reliability” or “content validity” of the intervention program. These terms may be stronger than the present design allows. The study demonstrates systematic development, expert appraisal, and refinement of the program, but it does not appear to include a formal reliability assessment or a conventional content-validity analysis. I therefore suggest replacing “reliability” with wording such as “systematic refinement and expert appraisal,” and replacing “content validity” with “perceived relevance, clarity, and appropriateness,” unless formal content-validity indices were calculated.

It would also be helpful to ensure that the Conclusion continues to maintain the same cautious distinction used elsewhere in the manuscript: the program is a structured and evidence-informed framework ready for caregiver-informed feasibility testing, rather than an intervention whose feasibility or effectiveness has already been established.

Subject to these minor wording revisions and a final technical check of the clean manuscript and supporting files, I believe the manuscript is suitable for publication.

[Note: HTML markup is below. Please do not edit.]

Reviewers' comments:

Reviewer's Responses to Questions

**Comments to the Author**

1. If the authors have adequately addressed your comments raised in a previous round of review and you feel that this manuscript is now acceptable for publication, you may indicate that here to bypass the “Comments to the Author” section, enter your conflict of interest statement in the “Confidential to Editor” section, and submit your "Accept" recommendation.

Reviewer #2: All comments have been addressed

**********

2. Is the manuscript technically sound, and do the data support the conclusions?

The manuscript must describe a technically sound piece of scientific research with data that supports the conclusions. Experiments must have been conducted rigorously, with appropriate controls, replication, and sample sizes. The conclusions must be drawn appropriately based on the data presented.

Reviewer #2: Yes

**********

3. Has the statistical analysis been performed appropriately and rigorously?

Reviewer #2: Yes

**********

4. Have the authors made all data underlying the findings in their manuscript fully available?

The PLOS Data policy requires authors to make all data underlying the findings described in their manuscript fully available without restriction, with rare exception (please refer to the Data Availability Statement in the manuscript PDF file). The data should be provided as part of the manuscript or its supporting information, or deposited to a public repository. For example, in addition to summary statistics, the data points behind means, medians and variance measures should be available. If there are restrictions on publicly sharing data—e.g. participant privacy or use of data from a third party—those must be specified.

Reviewer #2: Yes

**********

5. Is the manuscript presented in an intelligible fashion and written in standard English?

PLOS ONE does not copyedit accepted manuscripts, so the language in submitted articles must be clear, correct, and unambiguous. Any typographical or grammatical errors should be corrected at revision, so please note any specific errors here.

Reviewer #2: Yes

**********

6. Review Comments to the Author

Please use the space provided to explain your answers to the questions above. You may also include additional comments for the author, including concerns about dual publication, research ethics, or publication ethics. (Please upload your review as an attachment if it exceeds 20,000 characters)

Reviewer #2: I am satisfied with the changes, clarifications, and the point-by-point responses. Most importantly, it refers to the ACCORD (ACcurate COnsensus Reporting Document) guideline from the EQUATOR Network.

**********

7. PLOS authors have the option to publish the peer review history of their article (what does this mean?). If published, this will include your full peer review and any attached files.

If you choose “no”, your identity will remain anonymous but your review may still be made public.

**Do you want your identity to be public for this peer review?**  For information about this choice, including consent withdrawal, please see our Privacy Policy.

Reviewer #2: No

**********

[NOTE: If reviewer comments were submitted as an attachment file, they will be attached to this email and accessible via the submission site. Please log into your account, locate the manuscript record, and check for the action link "View Attachments". If this link does not appear, there are no attachment files.]

To ensure your figures meet our technical requirements, please review our figure guidelines: https://journals.plos.org/plosone/s/figures

You may also use PLOS’s free figure tool, NAAS, to help you prepare publication quality figures: https://journals.plos.org/plosone/s/figures#loc-tools-for-figure-preparation.

NAAS will assess whether your figures meet our technical requirements by comparing each figure against our figure specifications.

# Author comment: Author response to Decision Letter 2

**DOI:** 10.1371/journal.pone.0354823.r004

12 Jul 2026

Response to Reviewers

Manuscript ID: PONE-D-26-16776R1

Title: An evidence-informed psychosocial intervention program for caregiver burden among family caregivers of children with autism spectrum disorder: a multi-stage development and Delphi study

Dear Dr. Jiang and Reviewer,

We sincerely thank the Academic Editor and Reviewer #2 for the careful evaluation of our revised manuscript and for the constructive comments that have helped us further clarify the scope and contribution of the study. We are grateful that Reviewer #2 considered the previous comments to have been adequately addressed. In this revision, we have focused on the remaining conceptual issue raised by the Academic Editor regarding the interpretation of the Delphi findings and the distinction between program development and formal validation or demonstrated feasibility/effectiveness.

All changes have been made in the revised manuscript and are shown in the tracked-changes version. A clean version of the manuscript is also provided.

Response to Academic Editor

Comment 1

The revised manuscript is substantially improved and now presents a clearer and more appropriately cautious account of the intervention-development process. The authors have responded carefully to the concerns raised about the earlier quantitative and qualitative phases, the interpretation of the Delphi findings, caregiver involvement, implementation planning, and the distinction between program development and demonstrated feasibility or effectiveness.

I particularly appreciate that the revised manuscript now describes Kendall’s W as modest, acknowledges that caregivers did not directly review the final post-Delphi program, and clearly states that the ongoing pilot data are not included in the present paper. These revisions make the scope and contribution of the study much more transparent.

Response:

We sincerely thank the Academic Editor for this positive and constructive assessment. We appreciate the recognition that the revised manuscript now presents a clearer and more cautious account of the intervention-development process, including the modest Kendall’s W values, the indirect nature of caregiver involvement, and the distinction between the present development study and ongoing pilot work. These points have been carefully retained in the current revision.

Comment 2

I have only one remaining conceptual concern. In several places, the manuscript still appears to suggest that the evidence synthesis and Delphi process support the “reliability” or “content validity” of the intervention program. These terms may be stronger than the present design allows. The study demonstrates systematic development, expert appraisal, and refinement of the program, but it does not appear to include a formal reliability assessment or a conventional content-validity analysis. I therefore suggest replacing “reliability” with wording such as “systematic refinement and expert appraisal,” and replacing “content validity” with “perceived relevance, clarity, and appropriateness,” unless formal content-validity indices were calculated.

Response:

Thank you for this important clarification. We fully agree that the present study did not include a formal reliability assessment or a conventional content-validity analysis, and that the evidence synthesis and Delphi process should not be interpreted as establishing the reliability or formal content validity of the intervention program.

In response, we carefully reviewed the manuscript and revised wording that could imply stronger methodological claims than the design supports. We now describe the contribution of the evidence synthesis and Delphi consultation as systematic development, expert appraisal, and refinement of the program, rather than as evidence of reliability or formal validity.

Specifically, we revised the relevant wording in the Abstract, Discussion, and Conclusion. The manuscript now refers to the Delphi findings as reflecting experts’ perceptions of the relevance, clarity, and appropriateness of the program content, rather than as establishing formal content validity.

For example, the Results section of the Abstract has been revised to state that the Delphi process contributed to systematic refinement and expert appraisal of the program and reflected experts’ perceptions of the relevance, clarity, and appropriateness of its content, while practical feasibility remains to be tested directly with caregivers.

In the Discussion, we revised the interpretation of the Delphi findings to clarify that the ratings should be understood as expert perceptions rather than evidence of formal content validity, strong consensus, feasibility, or effectiveness.

Comment 3

It would also be helpful to ensure that the Conclusion continues to maintain the same cautious distinction used elsewhere in the manuscript: the program is a structured and evidence-informed framework ready for caregiver-informed feasibility testing, rather than an intervention whose feasibility or effectiveness has already been established.

Response:

We agree and have revised the Conclusion to maintain this cautious distinction more explicitly. The revised Conclusion now states that the program is a structured, theory-driven, and evidence-informed framework developed for subsequent caregiver-informed feasibility testing. We also explicitly state that the Delphi process enabled systematic refinement and expert appraisal, but did not establish formal content validity, feasibility, or effectiveness.

The revised Conclusion further emphasizes that the framework is ready for direct caregiver-informed feasibility testing, including assessment of acceptability, delivery fidelity, participant burden, cultural fit, and emotional safety, before subsequent effectiveness evaluation.

Response to Reviewer #2

Comment

All comments have been addressed.

Response:

We sincerely thank Reviewer #2 for confirming that the previous comments have been adequately addressed and for the positive assessment of the revised manuscript. We also appreciate the reviewer’s recognition of the inclusion of the ACCORD guideline. No additional revisions were requested by Reviewer #2 in this round.

We thank the Academic Editor and Reviewer again for their time and constructive feedback. We hope that the revised manuscript now adequately addresses the remaining concerns and meets the publication criteria of PLOS ONE.

Sincerely,

Xiuchuan Yan, PhD

On behalf of all authors

Attachment Submitted filename: Response to Reviewers R2.docx

# Editor report: Decision Letter 2

**DOI:** 10.1371/journal.pone.0354823.r005

**Authors:** Yilin Jiang

13 Jul 2026

An evidence-informed psychosocial intervention program for caregiver burden among family caregivers of children with autism spectrum disorder: a multi-stage development and Delphi study

PONE-D-26-16776R2

Dear Dr. Yan,

We’re pleased to inform you that your manuscript has been judged scientifically suitable for publication and will be formally accepted for publication once it meets all outstanding technical requirements.

Within one week, you’ll receive an e-mail detailing the required amendments. When these have been addressed, you’ll receive a formal acceptance letter and your manuscript will be scheduled for publication.

An invoice will be generated when your article is formally accepted. Please note, if your institution has a publishing partnership with PLOS and your article meets the relevant criteria, all or part of your publication costs will be covered. Please make sure your user information is up-to-date by logging into Editorial Manager at Editorial Manager® and clicking the ‘Update My Information' link at the top of the page. For questions related to billing, please contact billing support.

If your institution or institutions have a press office, please notify them about your upcoming paper to help maximize its impact. If they’ll be preparing press materials, please inform our press team as soon as possible -- no later than 48 hours after receiving the formal acceptance. Your manuscript will remain under strict press embargo until 2 pm Eastern Time on the date of publication. For more information, please contact onepress@plos.org.

Kind regards,

Yilin Jiang

Academic Editor

PLOS One

# Editor report: Acceptance letter

**DOI:** 10.1371/journal.pone.0354823.r006

**Authors:** Yilin Jiang

PONE-D-26-16776R2

PLOS One

Dear Dr. Yan,

I'm pleased to inform you that your manuscript has been deemed suitable for publication in PLOS One. Congratulations! Your manuscript is now being handed over to our production team.

At this stage, our production department will prepare your paper for publication. This includes ensuring the following:

* All references, tables, and figures are properly cited

* All relevant supporting information is included in the manuscript submission,

* There are no issues that prevent the paper from being properly typeset

You will receive further instructions from the production team, including instructions on how to review your proof when it is ready. Please keep in mind that we are working through a large volume of accepted articles, so please give us a few days to review your paper and let you know the next and final steps.

Lastly, if your institution or institutions have a press office, please let them know about your upcoming paper now to help maximize its impact. If they'll be preparing press materials, please inform our press team within the next 48 hours. Your manuscript will remain under strict press embargo until 2 pm Eastern Time on the date of publication. For more information, please contact onepress@plos.org.

You will receive an invoice from PLOS for your publication fee after your manuscript has reached the completed accept phase. If you receive an email requesting payment before acceptance or for any other service, this may be a phishing scheme. Learn how to identify phishing emails and protect your accounts at https://explore.plos.org/phishing.

If we can help with anything else, please email us at customercare@plos.org.

Thank you for submitting your work to PLOS ONE and supporting open access.

Kind regards,

PLOS ONE Editorial Office Staff

on behalf of

Dr. Yilin Jiang

Academic Editor

PLOS One
